# Comparison of the Catalytic Performance of Several Coal Gangue-Based Catalysts on Tar-Rich Coal Pyrolysis in a TGA and a Fixed-Bed Reactor

**DOI:** 10.3390/nano16140869

**Published:** 2026-07-15

**Authors:** Zhibing Chang, Chao Wang, Zhiwei Hu, Chuchu Wang, Yuliang Ma, Huiyan Li, Mo Chu

**Affiliations:** School of Chemical and Environmental Engineering, China University of Mining and Technology (Beijing), Beijing 100083, China

**Keywords:** tar-rich coal, coal gangue, catalytic pyrolysis, tar, aromatic hydrocarbons

## Abstract

Coal gangue shows promise as a potential raw material for coal pyrolysis catalysts. This study prepared coal gangue char catalysts from two types of coal gangue (CGY and CGH) via pyrolysis at 700 °C under N_2_, O_2_/N_2_ and steam/N_2_ atmospheres, labeled as CGY-N, CGY-ON, CGY-SN and CGH-N, CGH-ON, CGH-SN, respectively. Their catalytic performance in tar-rich coal pyrolysis was evaluated using a thermogravimetric analyzer and a fixed-bed reactor. Results showed that reactive atmosphere-derived char catalysts promoted coal weight loss, with CGY-SN and CGH-SN increasing weight loss of coal pyrolysis at 600 °C from 20.51 wt% to 22.05 wt% and 22.50 wt%, respectively. These catalysts generally reduced tar yield while increasing semicoke and water yields. They also decreased monocyclic aromatic hydrocarbons and enriched polycyclic aromatic hydrocarbons in the tar, alongside promoting the production of CH_4_, H_2_ and CO. Furthermore, they enhanced the gasification reactivity of the resulting semicoke. CGY-ON demonstrated particularly high activity, which is likely associated with its richness in K-, Ca- and Fe-bearing minerals, coupled with the exposure of active sites resulting from organic matter consumption under the O_2_/N_2_ atmosphere. An exception was CGH-N and CGH-SN, which increased monocyclic aromatic hydrocarbons from 25.67% to 30.65% and 31.64%, possibly related to shape-selective catalysis in the newly formed micropores. These insights provide a preliminary basis for the development of efficient coal gangue-based catalysts for tar-rich coal pyrolysis.

## 1. Introduction

Tar-rich coal (TRC) is commonly defined as coal with a tar yield ranging from 7 wt% to 12 wt%. China possesses substantial reserves of TRC, with the five western provinces and autonomous regions alone holding an estimated 500 billion tons of total resources. This corresponds to a potential oil yield of approximately 50 billion tons and a gas resource of about 75 trillion cubic meters [1]. Establishing and advancing an industry focused on the exploitation and efficient conversion of TRC for the primary production of oil and gas is imperative. Such an initiative is not only crucial for diversifying domestic oil and gas supplies but also represents a vital pathway for achieving clean and efficient utilization of coal resources [2].

Pyrolysis is a key technology for converting tar-rich coal into tar and gas. However, it faces several challenges, including a high content of heavy tar components, difficulties in separating tar from dust and poor operational stability. The formation and subsequent condensation of these heavy tar fractions can lead to operational issues such as clogged pipes and inefficient gas–solid separation [3]. Catalytic pyrolysis presents a potential solution to these problems. The introduction of catalysts can modulate the pyrolysis process to favor the formation of lighter components, thereby improving tar quality [4]. Furthermore, catalytic pyrolysis facilitates the production of higher-value products, such as aromatic hydrocarbons, hydrogen and CH_4_, which has garnered significant research interest [5]. In the context of coal pyrolysis, high-quality tar is typically characterized by a high proportion of light fractions, low contents of oxygenates and heavy components, and a favorable distribution of aromatic hydrocarbons. Specifically, a tar enriched in monocyclic and bicyclic aromatic hydrocarbons (e.g., benzene, toluene, xylenes, and naphthalene derivatives) is desirable, as these compounds are valuable feedstocks for the chemical industry.

The primary categories of catalysts employed in coal pyrolysis currently encompass compounds of alkali and alkaline earth metals [6], metal salts [7], transition metals [8], zeolites [9,10], carbon-based materials [11,12], natural minerals [13,14] and industrial solid waste [15,16]. A significant research focus lies in the development of catalysts that exhibit high catalytic performance while maintaining low cost. Coal gangue (CG), a major solid waste (10–30% of coal output [17]), is a promising and cost-benign raw material for catalyst synthesis. It is rich in valuable mineral components, including major elements (Si, Al, Fe) and others (Ca, Mg, K, Na, plus trace rare elements), which underpin its potential for creating high-performance catalysts. CG and its derived char (CGC) have been employed as catalysts in several studies [18,19,20,21,22]. For instance, Liu et al. [18,19] employed both raw CG and CGC as catalysts in the pyrolysis of oily sludge to produce aromatic-rich oil and hydrogen-rich gas. Their findings indicated that CGC promotes the cracking of heavy oil and secondary reactions, thereby improving the quality of the oil. Moreover, CG-based catalysts were also used in the pyrolysis of pine [20], corncob [17] and waste plastics [21].

While CG-based catalysts have demonstrated efficacy in the pyrolysis of biomass, oil sludge and waste plastics, their application in the pyrolysis of TRC remains largely unexplored. Furthermore, although both raw CG and its pyrolyzed char have been employed as catalysts, a systematic comparison of their catalytic performance is lacking. Additionally, the inherent variability in CG composition leads to significant differences in the performance of the derived catalysts. Clarifying these issues is critical for the rational development and application of CG-based catalysts, yet no study to date has provided a comparative analysis of catalysts derived from different CG sources.

It should be noted that the catalytic activity of CG-based catalysts is inherently limited compared to well-established high-performance catalysts such as zeolites and supported metal oxides, which possess well-defined active sites and tailored pore structures. However, CG utilization as a catalyst precursor offers dual benefits: reducing environmental burdens associated with waste disposal and providing a cost-benign alternative for catalytic applications. Therefore, the primary objective of this study is not to pursue optimal catalytic performance, but rather to explore the feasibility and fundamental behavior of raw CG and its pyrolyzed char as low-cost catalysts in TRC pyrolysis. From this perspective, even moderate catalytic effects are of practical significance, as they demonstrate the potential of converting a waste stream into a functional material for coal conversion processes, thereby contributing to the principles of circular economy and sustainable development.

Based on the above discussion, this study employed two distinct CG samples as raw materials to prepare char catalysts via pyrolysis at 700 °C under different atmospheres: N_2_, O_2_/N_2_ and steam/N_2_. Both the raw CG samples and their derived char samples were utilized as catalysts in the catalytic pyrolysis of TRC, which was conducted in a thermogravimetric analyzer and a fixed-bed reactor. The catalytic performance was systematically evaluated based on pyrolysis weight loss characteristics, product distribution and the composition of pyrolytic products. This work aims to provide foundational insights for the development, preparation and application of CG-based catalysts in TRC pyrolysis.

## 2. Materials and Methods

### 2.1. Materials

The coal sample was collected from the Wutongzhuang Mine in Hebei Province, China. Two CG samples, obtained from the Yangshita Mine (Inner Mongolia Autonomous Region) and the Huiyang Mine (Shanxi Province), were designated as CGY and CGH, respectively. The coal sample was subjected to a Gray-King assay according to the Chinese standard GB/T 1341-2007 [22], yielding semicoke (77.84 wt%), tar (10.47 wt%), water (2.26 wt%) and gas (9.43 wt%). With a tar yield between 7 wt% and 12 wt%, the coal was classified as TRC. Proximate analysis of TRC, CGY and CGH was performed following the Chinese standard GB/T 212-2008 [23]. The ultimate analysis of TRC was conducted using an elemental analyzer (vario MACRO cube, Elementar, Langenfeld, Germany), with the results presented in Table 1. Furthermore, the elemental composition of the CG samples was determined by an X-ray fluorescence spectrometry (XRF-1800, Shimadzu, Kyoto, Japan), and the results are summarized in Table 2. The TRC and CG samples were pulverized to pass through an 0.18 mm sieve and air-dried for subsequent experiments. A portion of the CG was also crushed and sieved to a 6–13 mm particle size range for the pyrolysis experiments.

### 2.2. Preparation and Characterization of CG-Based Catalysts

To prepare the CG char catalysts, pyrolysis of the 6–13 mm CG particles was conducted using a setup illustrated in Figure S1, which has been described in detail in our previous work [24,25]. Briefly, approximately 50 g of CG was loaded into the reactor. The system was purged with N_2_ at a flow rate of 200 mL/min for 30 min to ensure an inert atmosphere. For pyrolysis under a pure N_2_ atmosphere, the flow was then adjusted to 100 mL/min. The temperature was raised from ambient to 700 °C at a heating rate of 5 °C/min, held at 700 °C for 60 min, and then allowed to cool to room temperature, after which the char sample was collected. Pyrolysis under O_2_/N_2_ and steam/N_2_ atmospheres was also performed. The procedure was identical to that under N_2_ until the temperature reached 700 °C. At this point, a gas stream of 100 mL/min O_2_ or 0.3 mL/min of water was introduced alongside the 100 mL/min N_2_ carrier gas and maintained throughout the 60 min holding period. All resulting char samples were subsequently ground to pass through an 0.18 mm sieve for further use. The chars derived from CGY under N_2_, O_2_/N_2_ and steam/N_2_ atmospheres were designated as CGY-N, CGY-ON and CGY-SN, respectively. Correspondingly, those from CGH were labeled as CGH-N, CGH-ON and CGH-SN.

X-ray diffraction patterns of the CG and char samples were obtained by using a powder X-ray diffractometer (D/max-2500PC, Rigaku, Tokyo, Japan) operated at 40 kV and 100 mA. The scans were performed over a 2θ range of 2.5–70° with a speed of 10°/min and a step size of 0.025°. Phase identification was carried out using the Jade 6.5 software package (Materials Data, Inc., Livermore, CA, USA). N_2_ adsorption–desorption isotherms were performed on an automatic surface and porosity analyzer (ASAP 2460, Micromeritics, Atlanta, GA, USA). Before the measurement, the catalyst sample was degassed at 200 °C for 6 h. NH_3_ temperature programmed desorption apparatus (AutoChem1 II 2920, Micromeritics, Norcross, GA, USA) was employed to analyze the acidity of CG-based catalysts. The procedure is as follows. First, about 80 mg sample was purged with nitrogen at a flow rate of 30 mL/min and heated from room temperature to 200 °C, which was held for 1 h. Afterwards, the sample was cooled to 50 °C and the gas was switched to NH_3_ at a flow rate of 30 mL/min and kept for 1 h. Then the gas was switched to helium at a flow rate of 30 mL/min and maintained for 0.5 h in order to remove the weak adsorbed NH_3_. Finally, the sample was heated to 700 °C at a heating rate of 10 °C/min to desorb NH_3,_ which was detected by TCD detector.

### 2.3. Thermogravimetric Analysis (TGA)

The pyrolysis and catalytic pyrolysis behaviors of the TRC were investigated using a thermogravimetric analyzer (TGA/DSC 1, Mettler-Toledo, Schwerzenbach, Switzerland). Both the coal and the CG-based catalysts were ground to a particle size of less than 0.18 mm. For each experiment, approximately 10 mg of sample was heated from 30 to 900 °C at a rate of 10 °C/min under a high-purity N_2_ atmosphere with a flow rate of 50 mL/min. In the catalytic pyrolysis tests, the tar-rich coal and the catalyst were uniformly mixed at a mass ratio of 4:1. This mixture was then ground for 10 min in a ceramic mortar to ensure homogeneity, before being subjected to thermogravimetric analysis. In light of the relatively low catalytic activity of the CG-based catalyst, a relatively high catalyst dosage was adopted to make the catalytic effect apparent, thereby allowing for further exploration.

### 2.4. Fixed-Bed Pyrolysis Experiments

**Figure 1:**
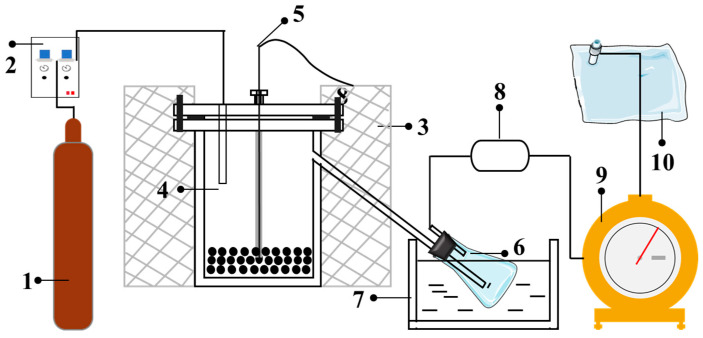
Schematic diagram of the experimental pyrolysis system. (1. N_2_ cylinder; 2. Mass flow controller; 3. Electric-ring furnace; 4. Stainless steel reactor; 5. Thermocouple; 6. Conical flask; 7. Ice-water bath; 8. Drying tube; 9. Wet gas flow meter; 10. Gas sampling bag). Reprinted from ref. [26].

A laboratory-scale fixed-bed reactor (Figure 1), constructed from stainless steel with dimensions of 950 mm in height and 46 mm in diameter, was employed for the pyrolysis experiments. The reactor, housed within an electric furnace, was equipped with a thermocouple embedded in the coal bed for precise temperature monitoring and control. High-purity N_2_ was introduced from the top of the reactor at a flow rate regulated by a mass flow controller. For non-catalytic pyrolysis tests, approximately 50 g of TRC (<0.18 mm) was loaded into the reactor. The system was first purged with N_2_ to establish an inert atmosphere. The temperature was then increased from ambient to target values of 400, 450, 500, 550 and 600 °C at a heating rate of 5 °C/min, held at each target temperature for 30 min, respectively.

The volatile products carried by the N_2_ stream were first directed into an ice-water cooled condenser to collect the majority of the tar and water. The remaining vapors were subsequently trapped by a tube packed with silica gel and cotton. The non-condensable gas volume was measured using a wet gas flow meter before collection for chromatographic analysis. The connections between the conical flask and the drying tube, and between the drying tube and the wet gas flow meter, were made using silicone tubing with an outer diameter of 8 mm, each with a length of approximately 300 mm. After the reaction, the system was cooled and the solid residue (semicoke) was weighed. The liquid condensate consisting of tar and water was first heated at 60 °C for 5 min to improve its fluidity. It was then transferred to a centrifuge tube. Phase separation of tar and water was achieved by centrifugation. The volume of the aqueous phase was measured directly to determine its mass. The tar mass was calculated by subtracting the mass of water from the total mass of the liquid mixture. The gas yield was determined by mass balance, and all product yields were calculated relative to the initial mass of the coal feedstock.

It should be noted that the weight gain of the drying tube in each experimental run was approximately 0.2 g, with minimal variation across runs. Given the difficulty in precisely determining the individual masses of tar and water contributing to this gain, and considering that this value is negligible compared to the mass of the tar-water mixture in the flask (ranging from 4.5 to 5 g for 550 °C pyrolysis experiments), this weight gain was attributed to the total gas mass. Additionally, some polar organic compounds (e.g., phenols) may partially dissolve in the aqueous phase during tar–water separation, which is a recognized limitation in coal tar analysis. Although the aqueous phase was not analyzed in this study, the small water volume (2.6–4.9 mL) and the dominance of the tar phase in the liquid products suggest that phenolic loss to water would not significantly affect the overall tar composition trends. Each set of experiments was conducted twice, and the product yields were calculated as the average of the two replicate experiments.

The results from these non-catalytic pyrolysis experiments indicated that the highest tar yield was achieved at 550 °C. Consequently, all subsequent catalytic pyrolysis experiments were conducted at 550 °C. For catalytic pyrolysis, the tar-rich coal was uniformly blended with the CG-based catalyst at a 4:1 mass ratio. The mixture was then ground in a ceramic mortar for 10 min to ensure homogeneity. All other experimental parameters were kept identical to those used in the non-catalytic runs.

### 2.5. Pyrolytic Product Analysis

Chemical analysis of the tar was performed by a gas chromatograph–mass spectrometer (Trace 1300-ISQ, Thermo Fisher Scientific, Waltham, MA, USA) equipped with a TG-5MS column (30 m × 0.25 mm ID × 0.25 μm). Briefly, the tar sample was first dissolved in chloroform to achieve a concentration of approximately 10 mg/mL. An aliquot of 1 μL of this solution was then injected in split mode (split ratio 50:1) with the injector temperature set at 280 °C. The temperature program for the GC oven began with a 3 min hold at 40 °C, followed by a ramp to 280 °C at 5 °C/min and a final hold for 50 min. The MS transfer line and ion source were maintained at 280 °C and 200 °C, respectively. Data were acquired after a 3 min solvent delay, scanning a mass range of 40–600 *m*/*z*. Compound identification was achieved by comparing the acquired mass spectra against the NIST mass spectral library.

The composition of the gaseous products was determined using a gas chromatography (GC-8600, Beifentianpu Instrument, Beijing, China). The analysis employed two separate columns: a packed 5A molecular sieve column (4.5 m × 2 mm ID) held at 50 °C for separating and quantifying CH_4_, H_2_ and CO via a thermal conductivity detector (TCD), and a packed silica gel column (1 m × 2 mm ID) operated under identical conditions for CO_2_ analysis. The volume fractions of these four gases (CH_4_, H_2_, CO CO_2_) were quantified using the external standard method. Subsequently, the yield of each gas component was calculated based on its measured volume fraction and the total volume of gas produced.

The gasification reactivity of the derived semicoke samples were investigated by using the thermogravimetric analyzer (TGA/DSC 1, Mettler-Toledo, Schwerzenbach, Switzerland). Briefly, about 5 mg semicoke sample was heated from 30 to 1000 °C at a rate of 10 °C/min under a CO_2_ atmosphere with a flow rate of 50 mL/min. The gasification reactivity of semicoke samples was explored by comparing their weight loss characteristics. The morphology of semicoke samples was assessed using a ZEISS Gemini SEM 300 scanning electron microscope (ZEISS, Oberkochen, Germany).

## 3. Results and Discussion

### 3.1. Characterization of CG-Based Catalysts

**Figure 2:**
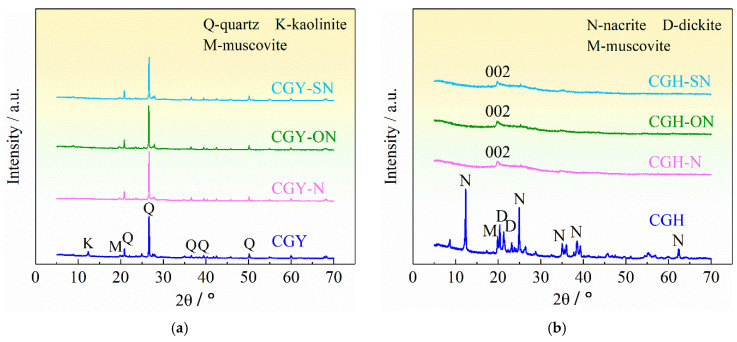
XRD patterns of CGY, CGH and their derived char samples. (**a**) CGY and derived char samples. (**b**) CGH and derived char samples.

Table 1 shows that CGY has a higher ash content than CGH, whereas CGH contains more volatile matter and fixed carbon, indicating a greater abundance of organic matter in CGH. Table 2 reveals that the primary chemical components of both CG samples are SiO_2_ and Al_2_O_3_. However, CGH has a significantly higher Al_2_O_3_ content, resulting in a lower SiO_2_/Al_2_O_3_ ratio (2.21) compared to that of CGY (5.13). The mineral composition presented in Figure 2 further demonstrates that CGY is predominantly composed of quartz, kaolinite and muscovite, while CGH mainly consists of nacrite, dickite and muscovite, consistent with the trend observed in the SiO_2_/Al_2_O_3_ ratios. Kaolinite has been reported to enhance tar yield during coal pyrolysis, which may be attributed to its active sites that stabilize generated free radicals to promote tar formation and facilitate the release of tar precursors [13]. Table 2 also indicates that CGY contains higher amounts of CaO and Fe_2_O_3_ compared to CGH. Both components are known to exhibit catalytic effects during pyrolysis [27,28]. For instance, CaO has been reported to promote the cleavage of oxygen-containing functional groups and facilitate the formation of aromatics through dehydrocyclization and aromatization pathways during catalytic upgrading of coal-derived volatiles [27]. Similarly, Song et al. [28] reported that Fe_2_O_3_ intensifies the pyrolysis process and enhances the cracking of oxygen-containing functional groups, directing their conversion toward CO.

Figure 2a shows that the characteristic diffraction peaks of kaolinite disappeared after pyrolysis at 700 °C, which aligns with the finding reported by Zou et al. [13] that the structure of kaolinite collapsed at temperatures above 500 °C. Furthermore, the nearly identical XRD patterns of the char samples obtained under different pyrolysis atmospheres suggest that the atmosphere has no significant influence on the mineral composition of the char samples. Figure 2b shows that most diffraction peaks of CGH disappeared after pyrolysis, attributable to the decomposition of these clay minerals. A broad hump observed at approximately 20° in the XRD patterns of char samples can be assigned to the two-dimensional (002) reflection, which corresponds to the interlayer spacing of aromatic structures [29]. This observation is consistent with the higher organic matter content in CGH. Similarly, the nearly identical diffraction patterns of CGH-derived char samples prepared under different atmospheres further confirm that the pyrolysis atmosphere has negligible impact on the crystalline phase composition.

Figure S2 presents the N_2_ adsorption–desorption isotherms of the raw CG and the resulting char samples. As shown in Figure S2a–d, the isotherms of CGY and its chars all conform to Type II, suggesting the absence of a well-developed pore structure in these samples. The corresponding pore structure parameters listed in Table 3 confirm that the porosity is predominantly mesoporous and macroporous, with both the micropore surface area (*S*_micro_) and micropore volume (*V*_micro_) being negligible, indicating a lack of microporosity. Furthermore, the isotherms exhibit H3-type hysteresis loops in the P/P_0_ range of 0.5 to 1.0, which is typically associated with the slit-shaped pores formed by the aggregation of plate-like particles. Table 3 also reveals that the specific surface area decreased after pyrolysis compared to the raw CGY, while the total pore volume showed no consistent trend, likely due to alterations in particle morphology during the thermal treatment.

Figure S2e–h displays the N_2_ adsorption–desorption isotherms of CGH and its derived char samples, which are also classified as Type II. In the initial stage of increasing P/P_0_, the N_2_ adsorption capacity of raw CGH rose sharply from 0 to approximately 2.5 cm^3^/g. In contrast, CGH-N and CGH-SN exhibited adsorption capacities close to or exceeding 5 cm^3^/g in the same region, suggesting the presence of micropores in CGH and their further development after pyrolysis. This is corroborated by the data in Table 3, which shows that *S*_micro_ and *V*_micro_ of CGH increased from 7.8 m^2^/g and 0.003 cm^3^/g to 13.0 m^2^/g and 0.006 cm^3^/g, respectively, after pyrolysis under N_2_. This implies that pyrolytic volatile created new micropores. Steam-assisted pyrolysis further enhanced *S*_micro_ and *V*_micro_ to 19.2 m^2^/g and 0.009 cm^3^/g, whereas pyrolysis under O_2_/N_2_ led to a reduction in *S*_micro_. This difference can be attributed to the slower gasification rate of steam, which allows it to penetrate and react within the organic matrix, thereby exerting a pore-opening effect. In contrast, the rapid surface oxidation by O_2_ consumed the organic matter more aggressively, resulting in a slight decrease in microporous area.

The NH_3_-TPD patterns of CG-based catalysts are shown in Figure S3. It reveals that distinct signal peaks appear for CGY and CGH in the range of 400–700 °C. This is primarily attributed to interference in the TCD signal caused by the release of crystalline water during kaolinite decomposition. In contrast, the CG-derived chars have already undergone pyrolysis at 700 °C and are not affected by such mineral decomposition. Based on Figure S3, the quantity and distribution of acid sites in the CG-derived chars were determined through peak area integration, and the results are presented in Table 4. The integration results for CGY and CGH are not listed because they are unreliable due to the crystalline water interference. The results indicate that no acid sites were detected in CGY-N, while the acid site quantity for CGH-N was 0.022 mmol/g. Introducing O_2_ or steam increased the acid site quantity to above 0.04 mmol/g. The likely reason is that oxidation and water-gas reactions generated more unsaturated carbon centers at the edges of the residual carbon skeleton, thereby contributing to Lewis acid sites. In addition, the etching of the carbon matrix under reactive atmospheres may also expose more acid sites originating from the mineral matrix.

### 3.2. Thermogravimetric Analysis of Catalytic Pyrolysis

**Figure 3:**
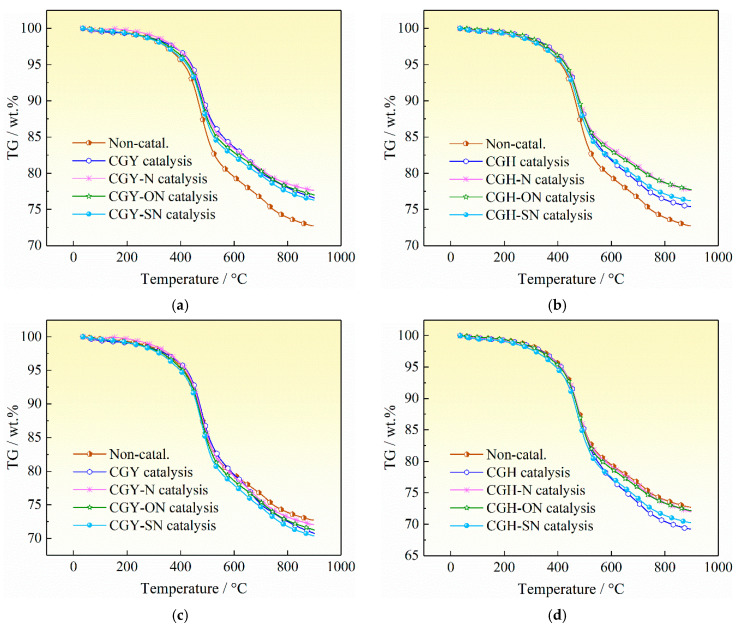

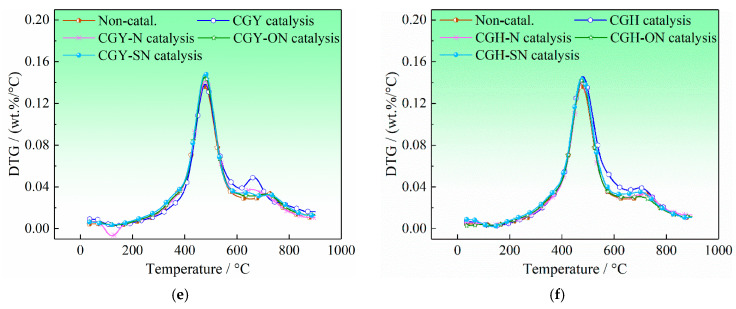
TG and DTG curves of catalytic pyrolysis of TRC. (**a**) Raw TG curves of TRC catalyzed by CGY-based catalysts. (**b**) Raw TG curves of TRC catalyzed by CGH-based catalysts. (**c**) Converted TG curves of TRC catalyzed by CGY-based catalysts. (**d**) Converted TG curves of TRC catalyzed by CGH-based catalysts. (**e**) Converted DTG curves of TRC catalyzed by CGY-based catalysts. (**f**) Converted DTG curves of TRC catalyzed by CGH-based catalysts.

Figure 3a,b present the TG curves for the pyrolysis and catalytic pyrolysis of tar-rich coal. As observed, the addition of either CG or its derived char led to a reduction in total weight loss compared to non-catalytic pyrolysis. This is expected as both CG and char are predominantly composed of minerals, which exhibit less weight loss during pyrolysis than coal does. To isolate the catalytic effect of these materials on the pyrolysis behavior of the coal, the TG curves were recalculated after deducting the mass of CG or CG-derived char, assuming their mass remained constant during the pyrolysis process. The corrected TG curves are shown in Figure 3c,d. The DTG curves obtained by differentiating the TG data using OriginPro 2017 software (OriginLab Corporation, Northampton, MA, USA) are displayed in Figure 3e,f. It should be noted that CG itself underwent significant weight loss during the TG experiment. This loss is unavoidably attributed to the coal, thereby overestimating the coal’s actual weight loss. In contrast, the CG-derived char samples have been pyrolyzed at 700 °C and they experienced negligible weight loss below 700 °C. Consequently, the TG and DTG curves for char-catalyzed tests more accurately represent the true catalytic pyrolysis behavior of the coal.

As shown in Figure 3c–f, the TG and DTG curves of non-catalytic and catalytic pyrolysis do not overlap, indicating that both raw CG and CG-derived char influence the coal pyrolysis process. To further investigate the catalytic effects, key characteristic parameters including weight loss at 400 °C (Δ*w*_400_), weight loss at 600 °C (Δ*w*_600_) and the maximum weight loss rate (d*w*/d*T*)_max_ were extracted and are summarized in Table 5. The results show that char samples obtained under O_2_/N_2_ and steam/N_2_ atmospheres increase both Δ*w*_400_ and Δ*w*_600_, with a more pronounced effect observed for steam-treated samples. For example, CGY-SN and CGH-SN raised Δ*w*_400_ from 4.40 wt% to 5.06 wt% and 5.31 wt%, respectively, and increased Δ*w*_600_ from 20.51 wt% to 22.05 wt% and 22.50 wt%. In contrast, other CG-based catalysts showed negligible effects. Table 5 also indicates that the (d*w*/d*T*)_max_ of non-catalytic pyrolysis was 0.136 wt%/°C, which was enhanced in the presence of CG-based catalysts derived from O_2_/N_2_ and steam/N_2_ pyrolysis. For instance, under catalysis by CGY-ON and CGY-SN, the (d*w*/d*T*)_max_ reached to 0.146 and 0.149 wt%/°C, respectively. This enhancement is likely attributed to the exposure of more active sites in the CG during reactive-atmosphere pyrolysis. This observation is consistent with previous studies reporting that Fe_2_O_3_ can increase the (d*w*/d*T*)_max_ of coal pyrolysis [28].

### 3.3. Pyrolysis Products Distribution

**Figure 4:**
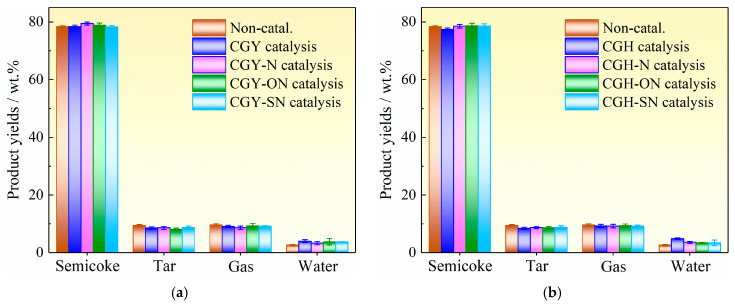
Product distribution of TRC pyrolysis catalyzed by CG-based catalysts. (**a**) CGY-based catalysts. (**b**) CGH-based catalysts.

Figure S4 shows the product distribution from the non-catalytic pyrolysis of tar-rich coal at different temperatures. As the pyrolysis temperature increased from 400 to 600 °C, the semicoke yield gradually decreased, while the yields of gas and water increased progressively. The tar yield initially rose and then declined, peaked at 9.40 wt% at 550 °C. Based on this observation, a temperature of 550 °C was selected for the subsequent catalytic pyrolysis experiments. Figure 4 illustrates the product yields from the catalytic pyrolysis of tar-rich coal. It should be emphasized that the CG-based catalysts, especially the raw samples CGY and CGH, underwent decomposition and thus experienced inevitable mass loss during pyrolysis. To specifically evaluate their catalytic effects, the results presented in Figure 4 were calculated under the simplifying assumption that the catalyst mass remained constant. Figure 4a shows that when CGY-based catalysts were applied, the tar yield and gas yield slightly decreased, while the yields of semicoke and water increased. Notably, CGY-ON exhibited a more pronounced effect, reducing the tar yield to 8.63 wt% and increasing the semicoke and water yields to 78.66 wt% and 3.35 wt%, respectively. These catalytic effects may be primarily associated with Fe- and Ca-containing minerals in the CG. For instance, studies have shown that both CaO and Fe_2_O_3_ can catalyze deoxygenation reactions [30,31]. According to the radical mechanism of coal pyrolysis, the removal of hydroxyl groups during deoxygenation may result in the formation of H_2_O by consuming active hydrogen species in the system, thereby reducing the availability of hydrogen radicals required for stabilizing other radical fragments [3]. Pyrolysis under O_2_/N_2_ atmosphere may remove part of the organic matter, exposing more active Fe and Ca sites and thus enhancing the aforementioned catalytic effects.

As for the CGH-based catalysts, Figure 4b reveals trends similar to those in Figure 4a: these catalysts decreased the tar yield and increased the water yield. In contrast, the effect of CGH was more pronounced. This is likely related to its clay mineral phases. Previous studies indicate that clay minerals such as kaolinite, montmorillonite and illite contain acidic sites within their interlayers [32]. These acidic sites can catalyze deoxygenation reactions and influence product distribution by altering hydrogen transfer pathways. Pyrolysis of CGH at 700 °C induced the decomposition of its clay minerals. For instance, kaolinite undergoes dehydroxylation to form metakaolin, leading to the destruction of acidic sites. Consequently, the catalytic effects of CGH-N, CGH-ON and CGH-SN are inferior to that of pristine CGH. It is also worth noting that the addition of CGH reduced the semicoke yield, which may be attributed to the weight loss resulting from the thermal decomposition of the clay minerals in CGH itself.

Compared with other types of catalysts, the CG-based catalysts in this study exhibited a relatively modest regulatory effect on tar yield (a reduction of approximately 0.5–1.3 wt%). This is considerably lower than that achieved by supported catalysts (e.g., Co-char reduced tar yield by approximately 2.5 wt% at a loading of 20% [4]) and zeolite-based catalysts (e.g., HZ-24 derived from ZSM-5 decreased liquid yield by approximately 2 wt% [5]). This difference is primarily attributable to the limited content of active components (such as Fe_2_O_3_ and CaO) in coal gangue, which are also highly dispersed within an inert matrix.

Upon comparing Table 5 and Figure 4, the results from thermogravimetric analysis and product distribution appear contradictory. As shown in Table 5, catalytic pyrolysis increased the weight loss at 600 °C, suggesting higher volatile yields. In contrast, Figure 4 shows that catalytic pyrolysis increased semicoke yield and decreased volatile products. This discrepancy can be rationalized by the difference in sample loading and the resulting extent of secondary reactions, as systematically discussed by Liu et al. [3]. In the TGA, only ~10 mg of sample was used; the volatiles generated are rapidly swept away and experience a very limited temperature increase (less than 10 °C according to Liu et al. [3]), which largely suppresses secondary reactions. Thus, the catalytic sites primarily influence primary pyrolysis, promoting volatile release, as reflected in the increased weight loss (Table 5). In the fixed-bed reactor (~50 g), however, volatiles inevitably pass through a high-temperature zone with a much longer residence time, conditions that promote secondary reactions. As volatiles contact the catalytic sites, deoxygenation consumes hydrogen radicals, and their deficiency promotes polymerization and condensation of aromatic intermediates, ultimately leading to higher semicoke yield and reduced tar and gas. This interpretation is consistent with the recognized understanding that volatile secondary reactions are highly sensitive to reactor configuration and operating conditions.

### 3.4. Analyses of Tar Samples

**Figure 5:**
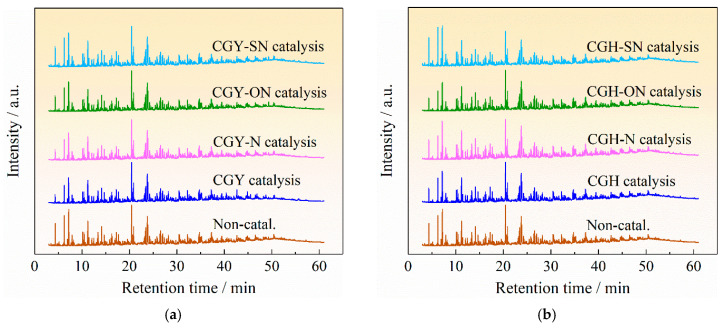
Total ion chromatograms of tar samples produced from TRC pyrolysis catalyzed by CG-based catalysts. (**a**) CGY-based catalysts. (**b**) CGH-based catalysts.

**Figure 6:**
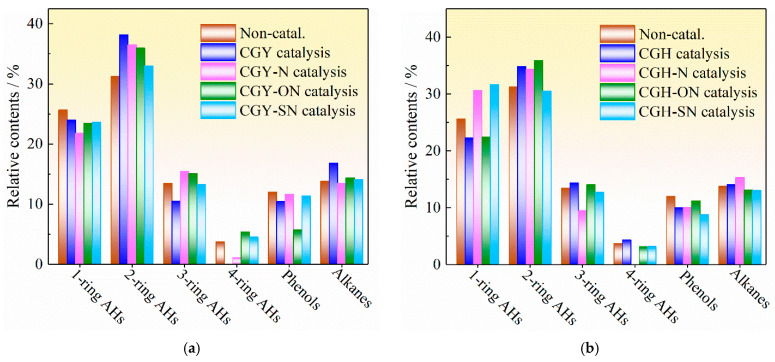
Relative contents of major components present in tar samples produced from TRC pyrolysis catalyzed by CG-based catalysts. (**a**) CGY-based catalysts. (**b**) CGH-based catalysts.

To investigate the influence of CG-based catalysts on the composition of coal tar, the tar samples were analyzed by GC–MS, with the total ion chromatograms (TIC) presented in Figure 5. By comparing the obtained mass spectra with the NIST database, the specific chemical composition of the tar was identified, which primarily consisted of aromatic hydrocarbons (AHs), phenols and alkanes. AHs were further classified based on the degree of ring condensation into monocyclic (1-ring), bicyclic (2-ring), tricyclic (3-ring) and tetracyclic (4-ring) AHs. The detailed identification results including retention times, compound names, relative contents and compound categories are summarized in Tables S1–S9. The relative content of each group is shown in Figure 6. It should be noted that the data presented here are semi-quantitative in nature, reflecting relative abundance rather than absolute concentrations. Therefore, changes in the relative proportions of compound groups should be interpreted with this limitation in mind.

Before evaluating the catalytic effects, it is instructive to examine the composition of tar obtained from non-catalytic pyrolysis. As shown in Figure 6, the tar is dominated by AHs, which account for over 70% of the total relative content. Among the AHs, 2-ring AHs are the most abundant (31.25%), followed by 1-ring AHs (25.67%), 3-ring AHs (13.47%) and 4-ring AHs (3.76%). Phenols and alkanes represent minor fractions, contributing 12.04% and 13.82%, respectively. This composition serves as a reference baseline against which the catalytic effects of CG-based catalysts on tar composition can be evaluated.

Figure 6a shows that when compared to non-catalytic pyrolysis, the CGY-based catalysts reduced the relative content of 1-ring AHs and phenols while enriching the proportion of polycyclic aromatic hydrocarbons (PAHs). This compositional shift is possibly related to catalytic condensation and ring fusion of aromatic structures, although the exact pathways remain to be elucidated. Liu et al. [18,19] also reported that CG char could catalyze dehydrogenation, cyclization, aromatization and polymerization during the pyrolysis of oily sludge, leading to the production of aromatic-rich oil. They attributed this catalytic effect to minerals such as kaolinite and boehmite. Among the various catalysts tested, CGY-ON demonstrated the most pronounced catalytic activity, reducing the relative phenol content from 12.04% to 5.75% and increasing the combined content of 3-ring and 4-ring AHs from 17.23% to 20.45%. Although phenolic loss to the aqueous phase cannot be fully ruled out, the systematic variation in phenol content across catalysts suggests that the observed reduction is mainly catalytic rather than an artifact. This enhanced performance is likely due to the consumption of partial organic matter under an oxidizing atmosphere, which exposed more active mineral sites and facilitated their contact with coal or volatile intermediates, thereby intensifying catalytic effects.

Figure 6b reveals that when compared to non-catalytic pyrolysis, both CGH and CGH-ON exhibit effects similar to those of CGY-based catalysts, namely reducing the content of 1-ring AHs and phenols while increasing the proportion of PAHs. In contrast, CGH-N and CGH-SN increased the relative abundance of 1-ring AHs from 25.67% to 30.65% and 31.64%, respectively. Considering that pyrolysis under N_2_ and steam/N_2_ increased the micropore volume from 0.003 cm^3^/g to 0.006 and 0.009 cm^3^/g, respectively (Table 3), it is hypothesized that newly formed micropores contain strong acidic sites. These acidic sites can promote the conversion of phenols to aromatics via deoxygenation and facilitate the transformation of alkanes into aromatics through dehydrogenation, cyclization and aromatization [33,34]. The shape-selective catalysis of the micropores likely surpassed the condensation and polymerization of aromatic rings, thereby contributing to the increased 1-ring AHs proportion. Aromatic hydrocarbons such as 1-ring, 2-ring and 3-ring types are valuable chemical feedstocks with broad applications. In recent years, the catalytic pyrolysis of carbonaceous fuels for aromatic production has attracted considerable research interest [5,6,18,19]. As illustrated in Figure 6, CGH-SN appears suitable for enriching 1-ring AHs on a relative basis whereas CGY-N is appropriate for generating 2-ring and 3-ring AHs.

### 3.5. Analyses of Pyrolytic Gases

**Figure 7:**
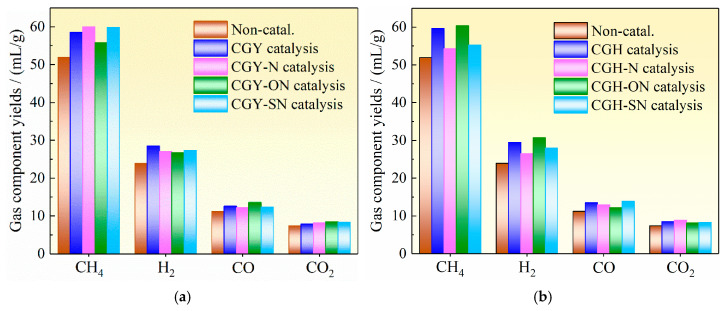
Yields of gas components derived from TRC pyrolysis catalyzed by CG-based catalysts. (**a**) CGY-based catalysts. (**b**) CGH-based catalysts.

Figure 7 presents the yields of gaseous components from the pyrolysis of tar-rich coal. The results demonstrate that CG-based catalysts enhance the production of both CH_4_ and H_2_. For instance, CGY-N increased CH_4_ and H_2_ yields from 51.9 and 23.9 mL/g to 60.1 and 27.0 mL/g, respectively. This enhancement can be attributed to the catalytic effect of the catalysts on the condensation of aromatic rings. During the condensation process, methyl and hydrogen attached to aromatic rings underwent cleavage, forming methyl and hydrogen radicals. The combination of methyl and hydrogen leads to the formation of CH_4_, while the combination of hydrogen radicals produces H_2_ [35]. Figure 7 also indicates that CG-based catalysts promoted the generation of CO and CO_2_. It is generally accepted that CO originates from the decarbonylation reaction, while CO_2_ is derived from decarboxylation during coal pyrolysis [36]. The increased yields of these gases suggest that the CG-based catalysts effectively facilitated these deoxygenation pathways.

### 3.6. Analyses of Semicoke Samples

**Figure 8:**
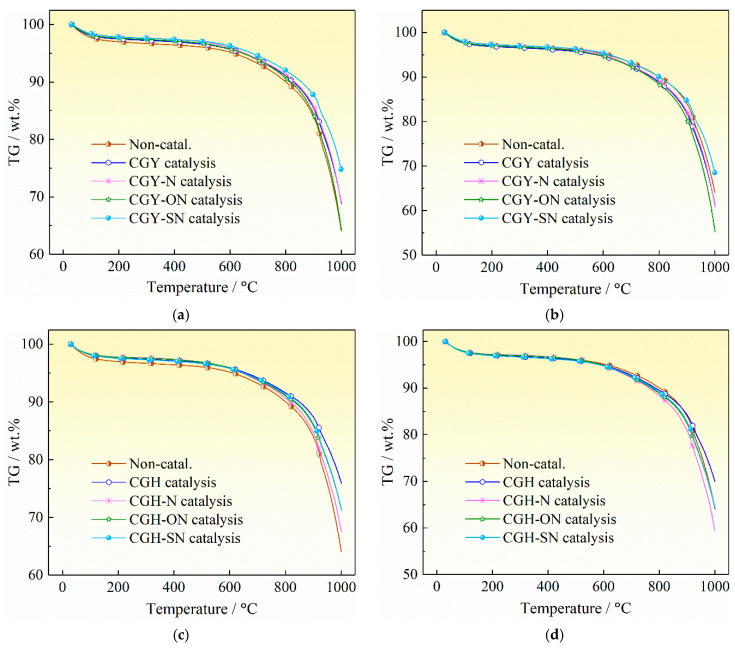
TG curves of CO_2_ gasification of semicoke samples derived from pyrolysis of TRC. (**a**) Raw TG curves of semicoke catalyzed by CGY-based catalysts. (**b**) Raw TG curves of semicoke catalyzed by CGH-based catalysts. (**c**) Converted TG curves of semicoke catalyzed by CGY-based catalysts. (**d**) Converted TG curves of semicoke catalyzed by CGH-based catalysts.

Figure 8a,b present the TG curves for the CO_2_ gasification of semicoke samples derived from the catalytic pyrolysis of TRC. It can be observed that the weight loss of the catalytic pyrolysis semicoke is consistently lower than that of the raw coal pyrolysis semicoke, which is attributed to the presence of CG-based catalysts within the semicoke. To investigate the effect of the CG-based catalysts on the gasification reactivity of the semicoke, the weight loss curves were recalculated after deducting the mass of the CG-based catalysts, assuming that these catalysts account for 25 wt% of the semicoke composition. The resulting curves are shown in Figure 8c,d. In fact, since the weight loss of coal during pyrolysis is greater than that of the CG-based catalyst, the actual proportion of the CG-based catalysts in the catalytic pyrolysis semicoke exceeds 25 wt%. Therefore, the results presented in Figure 8c,d still underestimate the CO_2_ gasification weight loss of the catalytic pyrolysis semicoke. Figure 8c shows that CGY-N and CGY-ON increased the weight loss at 1000 °C from 35.9 wt% to 39.2 wt% and 44.7 wt%, respectively. Figure 8d indicates that CGH-N increased this value to 40.6 wt%. These findings demonstrate that the CG-based catalysts enhance the CO_2_ gasification reactivity of the semicoke.

**Figure 9:**
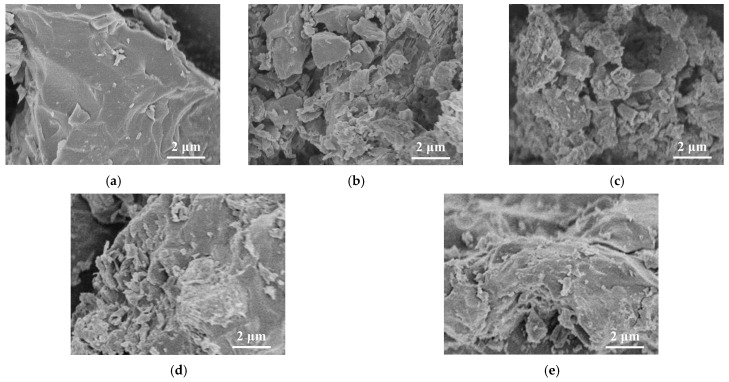
SEM images of semicoke samples derived from pyrolysis of TRC. (**a**) Non-catalytic pyrolysis. (**b**) CGY catalytic pyrolysis. (**c**) CGY-N catalytic pyrolysis. (**d**) CGY-ON catalytic pyrolysis. (**e**) CGY-SN catalytic pyrolysis.

Figure 9 presents the SEM images of the semicoke samples. It can be observed that the non-catalytic pyrolysis semicoke exhibits a relatively smooth surface with few fractures and pores (Figure 9a). In contrast, under catalytic pyrolysis conditions, the semicoke surface became rough and was prone to the adhesion of CG-based catalysts. The results in Figure 8 indicate that CGY-ON exhibits the most pronounced promoting effect on CO_2_ gasification. Given that CGY contains substantial amounts of K-, Ca- and Fe-bearing minerals, it is therefore inferred that the O_2_/N_2_ atmosphere during the preparation of CGY-ON may expose more active K, Ca and Fe sites, thereby catalyzing the CO_2_ gasification reaction [37].

### 3.7. Limitations and Implications of This Study

**Figure 10:**
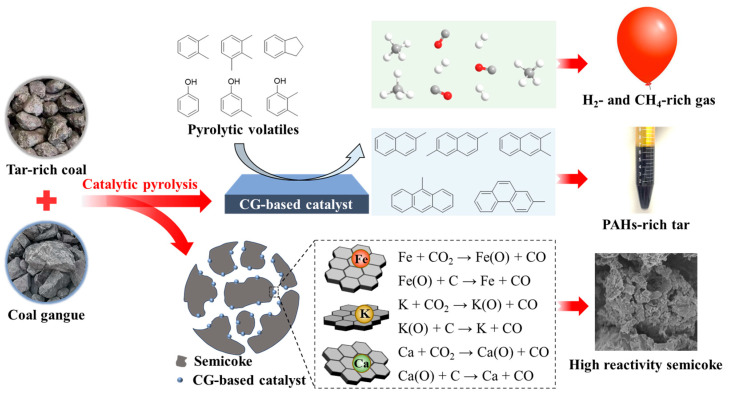
Main effects of CG-based catalysts on the composition and properties of products derived from TRC pyrolysis.

Despite their low BET surface areas (6–34 m^2^/g), the CG-based catalysts exhibit measurable catalytic activity in TRC pyrolysis. This activity appears to be governed primarily by the mineral composition rather than the porous structure, as further supported by the observation that CGY-ON showed the highest activity despite having the lowest surface area (6.0 m^2^/g). Porosity seems to play a secondary role in most cases, except for CGH-derived chars, where micropores developed during N_2_ or steam/N_2_ pyrolysis may have exerted a shape-selective effect favoring the formation of 1-ring AHs. Overall, the CG-based catalysts promoted bond cleavage during TGA, resulting in increased weight loss. In the fixed-bed reactor, they may have facilitated the transformation of 1-ring AHs and phenols, leading to their reduced relative content and a corresponding increase in PAHs in the tar, along with enhanced yields of CH_4_ and H_2_. They also improved the CO_2_ gasification reactivity of the resulting semicoke, with CGY-ON exhibiting the strongest effect, which is likely associated with its abundant K-, Ca- and Fe-containing active species. Figure 10 summarizes these main effects. These findings provide insights into the preparation and application of CG-based catalysts for TRC pyrolysis.

This study is limited to comparing the effects of two types of CG and their derived char samples on the product distribution and composition from the pyrolysis of tar-rich coal. Due to the restricted number of coal and CG samples, it was difficult to establish a definitive structure–activity relationship between the composition of CG-based catalysts and their catalytic performance. Although the catalysts exhibited certain catalytic effects, their performance remains slight and moderate. Future research could focus on modifying CG or CG-derived char samples, for instance, by loading metal active sites [21] or introducing acidic sites [38], to enhance catalytic activity. Alternatively, low-cost synthesis of various types of zeolites [39] from CG could be explored to evaluate their performance in TRC pyrolysis, thereby identifying suitable CG-based zeolite catalysts for this process.

## 4. Conclusions

This study prepared char samples from two types of coal gangue via pyrolysis at 700 °C under N_2_, O_2_/N_2_ and steam/N_2_ atmospheres. The catalytic effects of the raw coal gangue and their derived chars on the pyrolysis of tar-rich coal were systematically investigated using thermogravimetric analysis and a fixed-bed reactor. The main conclusions of this study include:

1. Thermogravimetric results indicated that chars obtained under reactive atmospheres showed a promoting effect on the pyrolysis of tar-rich coal, as evidenced by increases in both the weight loss rate and total weight loss. Specifically, CGY-SN and CGH-SN raised Δ*w*_600_ from 20.51 wt% to 22.05 wt% and 22.50 wt%, respectively, while increasing (d*w*/d*T*)_max_ from 0.136 wt%/°C to 0.149 and 0.145 wt%/°C, respectively.
2. The coal gangue-based catalysts generally reduced tar yield while increasing semicoke and water yields, decreased the content of 1-ring AHs and phenols, and promoted the formation of PAHs in tar. They also promoted the generation of valuable gases (e.g., CH_4_, H_2_ and CO) and enhanced the gasification reactivity of semicoke. CGY-ON exhibited the highest activity, which is likely associated with the exposure of active sites under the O_2_/N_2_ atmosphere that may facilitate polymerization and deoxygenation reactions.
3. Although coal gangue and its chars show moderate but promising catalytic potential for producing aromatics, hydrogen-rich gas and reactive semicoke from TRC pyrolysis, their overall effectiveness remains limited relative to established catalysts like zeolites and supported metal oxides. Future work should therefore focus on performance enhancement through strategies such as metal loading or the synthesis of coal gangue-based zeolites to improve activity and selectivity.

## Figures and Tables

**Table 1:** Proximate analysis and ultimate analysis of the tar-rich coal.

Sample	Proximate Analysis/(wt%, ad)				Ultimate Analysis/(wt%, daf)				
	M	A	V	FC	C	H	N	S	O *
TRC	0.96	8.65	29.08	61.32	88.15	6.08	1.71	0.28	3.78
CGY	2.39	84.12	8.22	5.27	-	-	-	-	-
CGH	1.28	74.83	16.11	7.78	-	-	-	-	-

* By difference.

**Table 2:** Main chemical composition of the CG samples.

Sample	Chemical Composition/wt%										
	SiO_2_	Al_2_O_3_	Fe_2_O_3_	K_2_O	Na_2_O	CaO	MgO	SO_3_	P_2_O_5_	Others	SiO_2_/Al_2_O_3_
CGY	64.64	21.43	3.56	3.59	0.87	2.51	0.92	0.30	0.18	2.00	5.13
CGH	52.37	40.36	0.58	1.36	0.26	0.07	0.62	2.98	0.03	1.37	2.21

**Table 3:** Textural properties of CG and derived char samples.

Catalyst	*S*_BET_ (m^2^/g) ^A^	*S*_micro_ (m^2^/g) ^B^	*S*_ext_ (m^2^/g) ^C^	*V*_total_ (cm^3^/g) ^D^	*V*_micro_ (cm^3^/g) ^B^	*V*_ext_ (cm^3^/g) ^C^	*D*_pore_ (nm) ^E^
CGY	10.0	0	10.0	0.044	0	0.044	3.40
CGY-N	8.7	0	8.7	0.058	0	0.058	3.81
CGY-ON	6.0	0	6.0	0.042	0	0.042	3.40
CGY-SN	8.3	0	8.3	0.046	0	0.046	3.81
CGH	22.4	7.8	14.6	0.038	0.003	0.035	4.30
CGH-N	29.1	13.0	16.1	0.026	0.006	0.020	3.81
CGH-ON	21.4	6.7	14.7	0.035	0.003	0.032	3.40
CGH-SN	33.7	19.2	14.5	0.024	0.009	0.015	3.05

^A^ Determined by multipoint BET method. ^B^ Measured by the t-plot method. ^C^ Calculated by difference. ^D^ Calculated from absorbed volume of N_2_ at a relative pressure P/P_0_ of 0.99. ^E^ Determined by BJH method using the adsorption branches of N_2_ isotherms.

**Table 4:** Acidity distribution of CG-based catalysts.

Catalyst	Acid Amount/(mmol/g)			Peak Position/°C	
	Weak ^A^	Strong ^A^	Total	Weak	Strong
CGY-N	- ^B^	- ^B^	- ^B^	- ^B^	- ^B^
CGY-ON	0.013	0.033	0.046	111	349
CGY-SN	0.004	0.041	0.045	105	434
CGH-N	- ^B^	0.022	0.022	- ^B^	307
CGH-ON	0.003	0.043	0.046	89	469
CGH-SN	0.001	0.041	0.042	80	438

^A^ Acid amount determined by integrated area of desorption peak in NH_3_-TPD. ^B^ Not detected.

**Table 5:** Characteristic parameters of TRC catalytic pyrolysis.

Parameter	Non-Catal.	Catalytic Pyrolysis							
		CGY	CGY-N	CGY-ON	CGY-SN	CGH	CGH-N	CGH-ON	CGH-SN
Δ*w*_400_/wt%	4.40	3.98	3.99	4.63	5.06	4.44	4.51	4.67	5.31
Δ*w*_600_/wt%	20.51	20.51	20.75	21.42	22.05	22.73	20.73	21.13	22.50
(d*w*/d*T*)_max_/ (wt%/°C)	0.136	0.139	0.142	0.146	0.149	0.146	0.141	0.142	0.145

## Data Availability

The original contributions presented in this study are included in the article/Supplementary Material. Further inquiries can be directed to the corresponding author.

## Supplementary Materials

The following supporting information can be downloaded at: https://www.mdpi.com/article/10.3390/nano16140869/s1, Figure S1: Schematic diagram of the experimental pyrolysis system used for preparation of CG-based catalysts; Figure S2: N2 adsorption–desorption isotherms of CG and derived char samples; Figure S3: NH_3_ temperature programmed desorption profiles of CG-based catalysts; Figure S4: Product distribution from non-catalytic pyrolysis of tar-rich coal at different temperatures; Table S1: Major components identified in tar from non-catalytic pyrolysis; Table S2: Major components identified in tar from catalytic pyrolysis by CGY; Table S3: Major components identified in tar from catalytic pyrolysis by CGY-N; Table S4: Major components identified in tar from catalytic pyrolysis by CGY-ON; Table S5: Major components identified in tar from catalytic pyrolysis by CGY-SN; Table S6: Major components identified in tar from catalytic pyrolysis by CGH; Table S7: Major components identified in tar from catalytic pyrolysis by CGH-N; Table S8: Major components identified in tar from catalytic pyrolysis by CGY-ON; Table S9: Major components identified in tar from catalytic pyrolysis by CGY-SN.

## Abbreviations

The following abbreviations are used in this manuscript:

TRC	Tar-rich coal
CG	Coal gangue
CGC	Coal gangue char
CGY	Coal gangue sampled from Yangshita coal mine
CGH	Coal gangue sampled from Huiyang coal mine
CGY-N	Char derived from pyrolysis of CGY under N_2_ atmosphere
CGY-ON	Char derived from pyrolysis of CGY under O_2_/N_2_ atmosphere
CGY-SN	Char derived from pyrolysis of CGY under steam/N_2_ atmosphere
CGH-N	Char derived from pyrolysis of CGH under N_2_ atmosphere
CGH-ON	Char derived from pyrolysis of CGH under O_2_/N_2_ atmosphere
CGH-SN	Char derived from pyrolysis of CGH under steam/N_2_ atmosphere
Δ*w*_400_	Weight loss at 400 °C from thermogravimetric analysis
Δ*w*_600_	Weight loss at 600 °C from thermogravimetric analysis
(d*w*/d*T*)_max_	Maximum weight loss rate from thermogravimetric analysis
TIC	Total ion chromatogram
AHs	Aromatic hydrocarbons
PAHs	Polycyclic aromatic hydrocarbons
