# Supplementary material for: Comparison of the Catalytic Performance of Several Coal Gangue-Based Catalysts on Tar-Rich Coal Pyrolysis in a TGA and a Fixed-Bed Reactor
Source: Nanomaterials (Basel). 2026 Jul 15;16(14):869. doi: 10.3390/nano16140869 (PMC13415308; doi:10.3390/nano16140869)
Supplement: Supplementary file 1 [file nanomaterials-16-00869-s001.zip › nanomaterials-4380158-supplementary.pdf]

Article

Comparison of the Catalytic Performance of Several Coal Gangue-Based Catalysts on Tar-Rich Coal Pyrolysis in a TGA and a Fixed-Bed Reactor

Zhibing Chang *, Chao Wang, Zhiwei Hu, Chuchu Wang, Yuliang Ma, Huiyan Li and Mo Chu

School of Chemical and Environmental Engineering, China University of Mining and Technology (Beijing), Beijing 100083, China
* Correspondence: achang515551103@163.com

Supporting Information

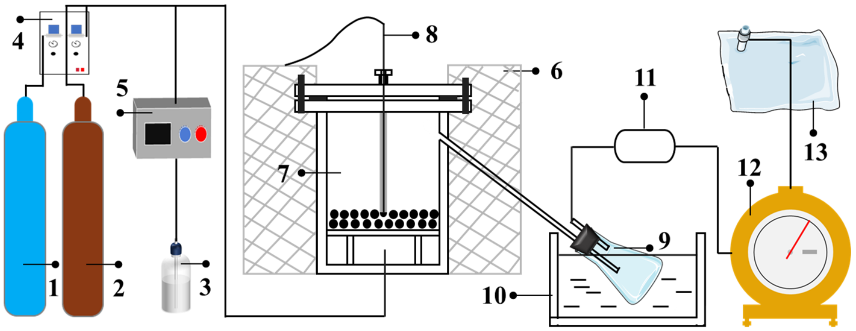

Figure S1. Schematic diagram of the experimental pyrolysis system used for preparation of CG-based catalysts. (1. O2 cylinder; 2. N2 cylinder; 3. Deionized water; 4. Mass flow controller; 5. Micro-peristaltic pump; 6. Electric-ring furnace; 7. Stainless steel reactor; 8. Thermocouple; 9. Conical flask; 10. Ice-water bath; 11. Drying tube; 12. Wet gas flow meter; 13. Gas sampling bag).

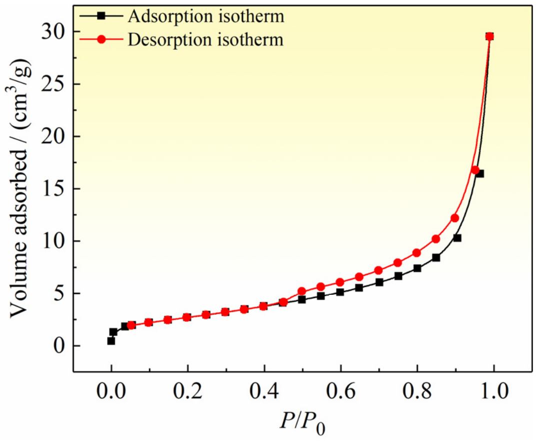
Adsorption isotherm
Desorption isotherm
Volume adsorbed / (cm^3/g)
P/P_0
0 5 10 15 20 25 30
0.0 0.2 0.4 0.6 0.8 1.0

(a)

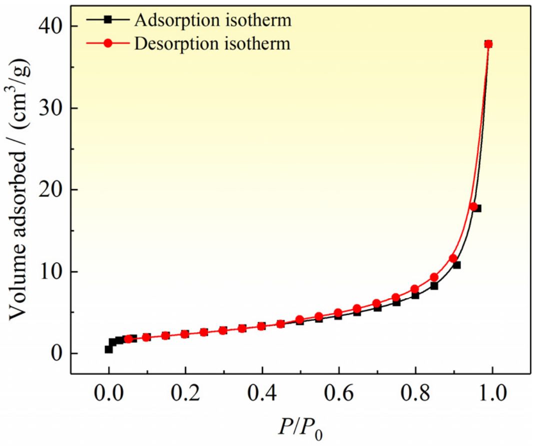
Adsorption isotherm
Desorption isotherm
Volume adsorbed / (cm^3/g)
P/P_0
0 10 20 30 40
0.0 0.2 0.4 0.6 0.8 1.0

(b)

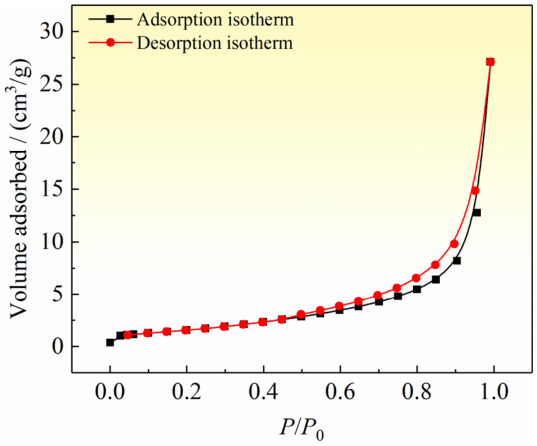
Adsorption isotherm
Desorption isotherm
Volume adsorbed / (cm^3/g)
P/P_0
0 5 10 15 20 25 30
0.0 0.2 0.4 0.6 0.8 1.0

(c)

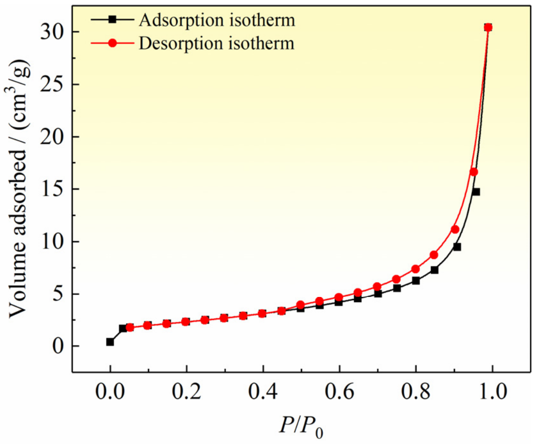
Adsorption isotherm
Desorption isotherm
Volume adsorbed / (cm^3/g)
P/P_0
0 5 10 15 20 25 30
0.0 0.2 0.4 0.6 0.8 1.0

(d)

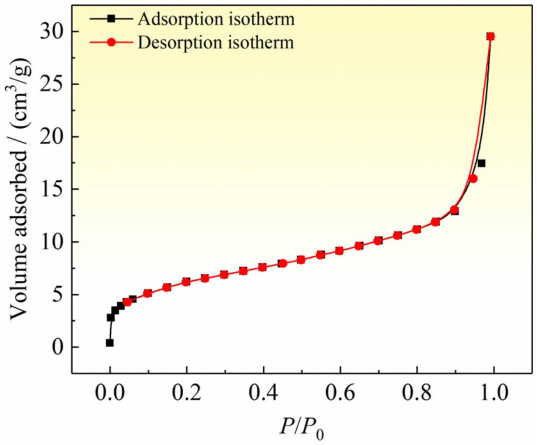
Adsorption isotherm
Desorption isotherm
Volume adsorbed / (cm^3/g)
P/P_0
0 5 10 15 20 25 30
0.0 0.2 0.4 0.6 0.8 1.0

(e)

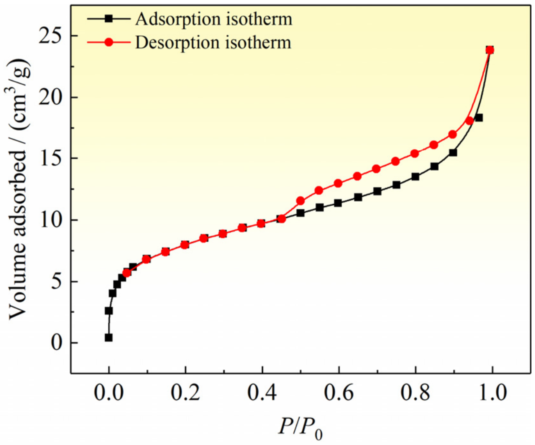
Adsorption isotherm
Desorption isotherm
Volume adsorbed / (cm^3/g)
P/P_0
0 5 10 15 20 25
0.0 0.2 0.4 0.6 0.8 1.0

(f)

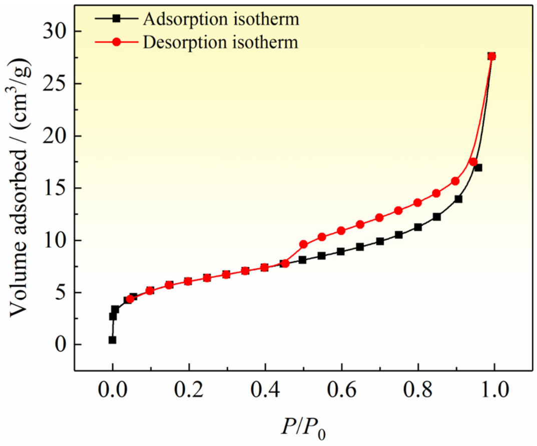

(g)

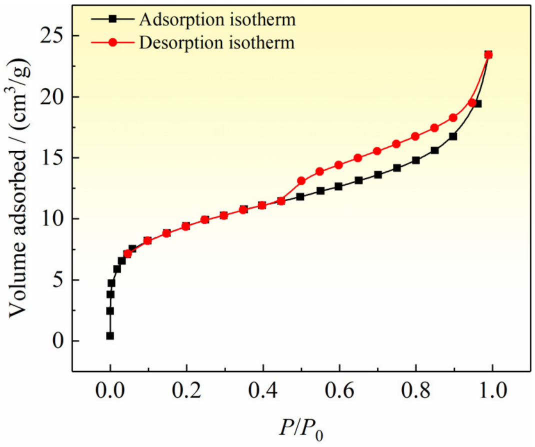

(h)

Figure S2. N_2 adsorption–desorption isotherms of CG and derived char samples. (a) CGY. (b) CGY-N. (c) CGY-ON. (d) CGY-SN. (e) CGH. (f) CGH-N. (g) CGH-ON. (h) CGH-SN.

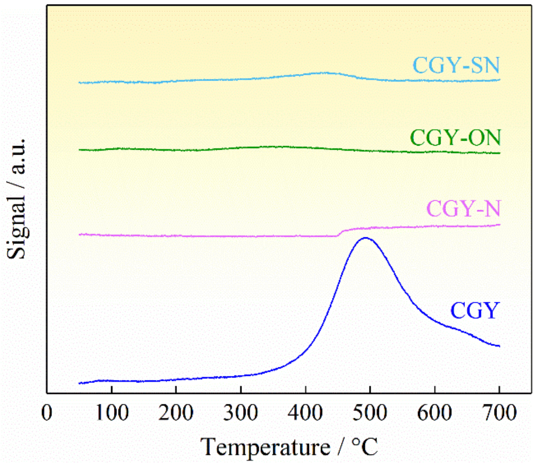

(a)

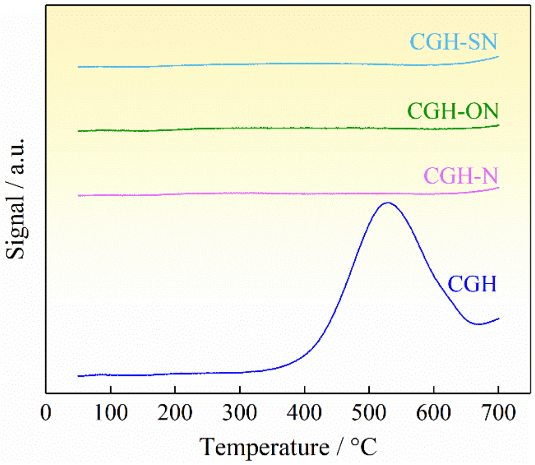

(b)

Figure S3. NH_3 temperature programmed desorption profiles of CG-based catalysts. (a) CGY and derived char samples. (b) CGH and derived char samples.

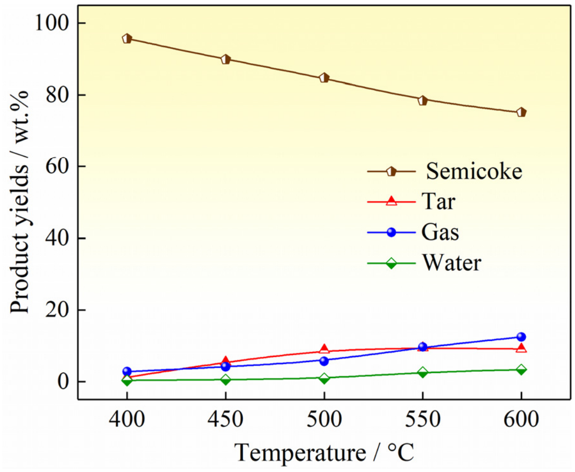

Figure S4. Product distribution from non-catalytic pyrolysis of tar-rich coal at different temperatures.

Table S1. Major components identified in tar from non-catalytic pyrolysis.

Number	Retention time (min)	Name	Relative content (%)	Category
1	4.339	Toluene	2.32	1-ring AHs
2	6.265	Cyclohexane, 1,1,3-trimethyl-	3.66	Alkanes
3	7.2	o-Xylene	5.1	1-ring AHs
4	7.242	Benzene, 1,3-dimethyl-	1.18	1-ring AHs
5	7.903	o-Xylene	1.58	1-ring AHs
6	10.149	Benzene, 1-ethyl-3-methyl-	1.79	1-ring AHs
7	10.431	Benzene, 1,2,4-trimethyl-	1.94	1-ring AHs
8	11.228	Benzene, 1,2,4-trimethyl-	5.38	1-ring AHs
9	11.49	Nonane, 2-methyl-	1.15	Alkanes
10	12.091	Benzene, 1,2,4-trimethyl-	0.97	1-ring AHs
11	13.28	Benzene, 2-ethyl-1,3-dimethyl-	1.06	1-ring AHs
12	13.406	Phenol, 3-methyl-	2.11	Phenols
13	14.142	Phenol, 3-methyl-	4.33	Phenols
14	14.678	Undecane	1.54	Alkanes
15	16.096	1H-Indene, 2,3-dihydro-4-methyl-	1.13	1-ring AHs
16	16.289	Phenol, 2,3-dimethyl-	2.09	Phenols
17	16.35	Phenol, 2,6-dimethyl-	1.2	Phenols
18	16.975	Phenol, 2,3-dimethyl-	1.05	Phenols
19	17.226	Azulene	1.88	2-ring AHs
20	17.717	Tridecane	1.29	Alkanes
21	18.875	Phenol, 2-ethyl-5-methyl-	1.04	Phenols
22	20.45	Naphthalene, 2-methyl-	6.07	2-ring AHs
23	20.572	Dodecane	1.38	Alkanes
24	20.866	Naphthalene, 1-methyl-	2.59	2-ring AHs
25	23.261	Dodecane	1.01	Alkanes
26	23.401	Naphthalene, 2,6-dimethyl-	2.28	2-ring AHs
27	23.451	Naphthalene, 2,6-dimethyl-	2.36	2-ring AHs
28	23.76	Naphthalene, 2,3-dimethyl-	4.83	2-ring AHs
29	23.867	Naphthalene, 2,6-dimethyl-	2.76	2-ring AHs
30	24.277	Naphthalene, 2,3-dimethyl-	1.8	1-ring AHs
31	26.4	Naphthalene, 1,4,6-trimethyl-	1.5	2-ring AHs
32	26.563	4,6,8-Trimethylazulene	2.05	2-ring AHs

33	26.902	Naphthalene, 1,4,6-trimethyl-	1.34	2-ring AHs
34	27.021	4,6,8-Trimethylazulene	1.63	2-ring AHs
35	27.818	Fluorene	0.96	1-ring AHs
36	28.183	Dodecane, 2-methyl-6-propyl-	1.46	Alkanes
37	30.456	3H-Benz[e]indene, 2-methyl-	2.08	Alkanes
38	30.58	9H-Fluorene, 2-methyl-	1.4	2-ring AHs
39	32.255	Phenanthrene	1.43	3-ring AHs
40	34.622	Anthracene, 9-methyl-	2.05	3-ring AHs
41	34.733	Phenanthrene, 1-methyl-	1.63	3-ring AHs
42	34.945	Naphtho[2,3-b]norbornadiene	1.28	3-ring AHs
43	37.045	Phenanthrene, 2,5-dimethyl-	1.07	3-ring AHs
44	37.283	Phenanthrene, 2,5-dimethyl-	2.61	3-ring AHs
45	37.417	Phenanthrene, 2,5-dimethyl-	1.9	3-ring AHs
46	39.481	Phenanthrene, 2,3,5-trimethyl-	1.26	3-ring AHs
47	40.98	11H-Benzo[b]fluorene	1.08	4-ring AHs
48	42.68	Pyrene, 1,3-dimethyl-	1.48	4-ring AHs
49	44.853	Cyclononasiloxane, octadecamethyl-	1.81	Impurity
50	46.552	Benz[a]anthracene, 7-methyl-	1.13	4-ring AHs

Table S2. Major components identified in tar from catalytic pyrolysis by CGY.

Number	Retention time (min)	Name	Relative content (%)	Category
1	4.328	Toluene	1.12	1-ring AHs
2	6.251	Cyclohexane, 1,1,3-trimethyl-	2.72	Alkanes
3	7.186	o-Xylene	4.22	1-ring AHs
4	7.233	o-Xylene	0.85	1-ring AHs
5	7.891	o-Xylene	1.42	1-ring AHs
6	8.194	Nonane	0.55	Alkanes
7	10.136	Benzene, 1,2,3-trimethyl-	1.9	1-ring AHs
8	10.242	Benzene, 1,2,3-trimethyl-	0.97	1-ring AHs
9	10.421	Benzene, 1,2,4-trimethyl-	2.1	1-ring AHs
10	11.216	Benzene, 1,2,4-trimethyl-	5.36	1-ring AHs
11	11.482	Decane	1.1	Alkanes
12	12.083	Benzene, 1,2,4-trimethyl-	1.05	1-ring AHs
13	12.505	Tetracyclo[3.3.1.0(2,8).0(4,6)]-non-2-ene	1.02	1-ring AHs
14	13.276	Benzene, 1-ethyl-2,3-dimethyl-	0.94	1-ring AHs
15	13.415	Phenol, 3-methyl-	2.31	Phenols
16	14.142	Phenol, 3-methyl-	4.62	Phenols
17	14.673	Undecane	1.62	Alkanes
18	15.817	1H-Indene, 2,3-dihydro-5-methyl-	0.65	1-ring AHs
19	16.089	1H-Indene, 2,3-dihydro-4-methyl-	0.94	1-ring AHs
20	16.291	Phenol, 2,5-dimethyl-	2.03	Phenols
21	16.358	Phenol, 2,6-dimethyl-	1.15	Phenols
22	17.216	Azulene	1.69	2-ring AHs
23	17.709	Dodecane	1.44	Alkanes
24	20.444	Naphthalene, 2-methyl-	7.67	2-ring AHs
25	20.567	Tridecane	1.62	Alkanes
26	20.858	Naphthalene, 1-methyl-	3.31	2-ring AHs
27	23.255	Dodecane	1.21	Alkanes
28	23.397	Naphthalene, 1,6-dimethyl-	2.76	2-ring AHs
29	23.446	Naphthalene, 2,6-dimethyl-	2.85	2-ring AHs
30	23.752	Naphthalene, 2,3-dimethyl-	5.98	2-ring AHs
31	23.863	Naphthalene, 2,6-dimethyl-	3.5	2-ring AHs
32	25.785	Pentadecane	1.04	Alkanes

33	26.395	Naphthalene, 1,4,6-trimethyl-	1.81	2-ring AHs
34	26.558	4,6,8-Trimethylazulene	2.22	2-ring AHs
35	26.895	Naphthalene, 1,4,6-trimethyl-	1.54	2-ring AHs
36	27.017	4,6,8-Trimethylazulene	1.83	2-ring AHs
37	27.313	3-(2-Methyl-propenyl)-1H-indene	0.61	1-ring AHs
38	28.178	Dodecane, 2-methyl-6-propyl-	1.84	Alkanes
39	30.45	Dodecane, 2-methyl-6-propyl-	2.28	Alkanes
40	30.572	9H-Fluorene, 2-methyl-	1.63	2-ring AHs
41	32.25	Phenanthrene	1.84	3-ring AHs
42	32.608	Hexadecane	0.79	Alkanes
43	34.613	Anthracene, 9-methyl-	2.52	3-ring AHs
44	34.725	Phenanthrene, 1-methyl-	2.08	3-ring AHs
45	35.083	1H-Cyclopropa[l]phenanthrene,1a,9b-dihydro-	2.78	3-ring AHs
46	37.274	Phenanthrene, 2,5-dimethyl-	0.92	3-ring AHs
47	37.908	Cyclononasiloxane, octadecamethyl-	0.72	Impurity
48	40.394	Cyclononasiloxane, octadecamethyl-	0.96	Impurity
49	42.681	Cyclononasiloxane, octadecamethyl-	0.81	Impurity
50	44.849	Cyclononasiloxane, octadecamethyl-	1.12	Impurity

Table S3. Major components identified in tar from catalytic pyrolysis by CGY-N.

Number	Retention time (min)	Name	Relative content (%)	Category
1	4.329	Toluene	1.18	1-ring AHs
2	6.251	Cyclohexane, 1,1,3-trimethyl-	2.57	Alkanes
3	7.187	o-Xylene	5.16	1-ring AHs
4	7.891	o-Xylene	1.34	1-ring AHs
5	10.138	Benzene, 1,2,3-trimethyl-	1.7	1-ring AHs
6	10.419	Benzene, 1,2,4-trimethyl-	1.78	1-ring AHs
7	11.219	Benzene, 1,2,4-trimethyl-	5.08	1-ring AHs
8	11.482	Undecane	1.2	Alkanes
9	12.083	Benzene, 1,2,4-trimethyl-	0.96	1-ring AHs
10	13.41	Phenol, 3-methyl-	1.89	Phenols
11	14.137	Benzene, 1-[(3-chloro-2-propenyl)oxy]-2-methyl-	4.3	1-ring AHs
12	14.672	Undecane	1.55	Alkanes
13	16.09	1H-Indene, 2,3-dihydro-5-methyl-	1.04	1-ring AHs
14	16.288	Phenol, 2,5-dimethyl-	1.89	Phenols
15	16.358	Phenol, 2,6-dimethyl-	1.03	Phenols
16	16.976	Phenol, 2,3-dimethyl-	0.94	Phenols
17	17.218	Azulene	1.92	2-ring AHs
18	17.711	Dodecane	1.4	Alkanes
19	20.443	Naphthalene, 2-methyl-	6.81	2-ring AHs
20	20.567	Dodecane	1.44	Alkanes
21	20.859	Naphthalene, 1-methyl-	2.84	2-ring AHs
22	23.255	Dodecane	1.16	Alkanes
23	23.394	Naphthalene, 2,6-dimethyl-	2.4	2-ring AHs
24	23.443	Naphthalene, 2,6-dimethyl-	2.52	2-ring AHs
25	23.751	Naphthalene, 2,3-dimethyl-	5.2	2-ring AHs
26	23.859	Naphthalene, 2,6-dimethyl-	2.94	2-ring AHs
27	24.271	Naphthalene, 2,3-dimethyl-	1.95	2-ring AHs
28	25.783	Pentadecane	0.9	Alkanes
29	25.916	Naphthalene, 1,4,6-trimethyl-	0.98	2-ring AHs
30	26.392	Naphthalene, 1,4,6-trimethyl-	1.58	2-ring AHs
31	26.555	4,6,8-Trimethylazulene	2.26	2-ring AHs
32	26.897	Naphthalene, 1,4,6-trimethyl-	1.41	2-ring AHs

33	27.015	4,6,8-Trimethylazulene	1.65	2-ring AHs
34	27.31	3-(2-Methyl-propenyl)-1H-indene	0.94	1-ring AHs
35	28.18	Dodecane, 2-methyl-6-propyl-	1.81	Alkanes
36	30.449	Decane, 1-bromo-2-methyl-	2.35	Alkanes
37	30.57	9H-Fluorene, 2-methyl-	1.49	2-ring AHs
38	32.249	Phenanthrene	1.62	3-ring AHs
39	34.611	Phenanthrene, 1-methyl-	1.68	3-ring AHs
40	34.724	Phenanthrene, 1-methyl-	1.95	3-ring AHs
41	34.936	1H-Cyclopropa[l]phenanthrene,1a,9b-dihydro-	1.48	3-ring AHs
42	35.078	1H-Cyclopropa[l]phenanthrene,1a,9b-dihydro-	0.88	3-ring AHs
43	37.032	Phenanthrene, 2,5-dimethyl-	1.14	3-ring AHs
44	37.27	Phenanthrene, 2,5-dimethyl-	2.89	3-ring AHs
45	37.433	Phenanthrene, 2,5-dimethyl-	1.99	3-ring AHs
46	39.469	Phenanthrene, 2,3,5-trimethyl-	1.46	3-ring AHs
47	40.964	Pyrene, 1-methyl-	1.19	4-ring AHs
48	42.671	Pyrene, 1,3-dimethyl-	1.54	4-ring AHs
49	44.684	Benz[a]anthracene	1.43	4-ring AHs
50	46.542	Chrysene, 1-methyl-	1.2	4-ring AHs

Table S4. Major components identified in tar from catalytic pyrolysis by CGY-ON.

Number	Retention time (min)	Name	Relative content (%)	Category
1	4.334	Toluene	1.86	1-ring AHs
2	6.259	Cyclohexane, 1,1,3-trimethyl-	3.22	Alkanes
3	7.195	o-Xylene	4.54	1-ring AHs
4	7.242	o-Xylene	1.2	1-ring AHs
5	7.898	o-Xylene	1.42	1-ring AHs
6	10.144	Benzene, 1-ethyl-3-methyl-	1.72	1-ring AHs
7	10.242	Benzene, 1,2,3-trimethyl-	0.84	1-ring AHs
8	10.426	Benzene, 1,2,4-trimethyl-	1.87	1-ring AHs
9	11.224	Benzene, 1,2,4-trimethyl-	5.25	1-ring AHs
10	11.486	Undecane	1.21	Alkanes
11	12.085	Benzene, 1,2,4-trimethyl-	0.96	1-ring AHs
12	12.509	Tetracyclo[3.3.1.0(2,8).0(4,6)]-non-2-ene	0.86	1-ring AHs
13	13.406	Phenol, 3-methyl-	2.06	Phenols
14	14.14	Phenol, 3-methyl-	4.14	Phenols
15	14.674	Undecane	1.53	Alkanes
16	16.093	1H-Indene, 2,3-dihydro-4-methyl-	1.01	1-ring AHs
17	16.286	Phenol, 2,5-dimethyl-	2.09	Phenols
18	16.35	Phenol, 2,6-dimethyl-	1.02	Phenols
19	16.973	Phenol, 2,5-dimethyl-	1.08	Phenols
20	17.221	Azulene	2.09	2-ring AHs
21	17.714	Tridecane	1.16	Alkanes
22	18.884	Phenol, 2-ethyl-5-methyl-	1.15	Phenols
23	20.449	Naphthalene, 2-methyl-	6.88	2-ring AHs
24	20.569	Dodecane	1.36	Alkanes
25	20.863	Naphthalene, 1-methyl-	2.89	2-ring AHs
26	23.257	Dodecane	1.06	Alkanes
27	23.399	Naphthalene, 2,6-dimethyl-	2.64	2-ring AHs
28	23.448	Naphthalene, 2,6-dimethyl-	2.32	2-ring AHs
29	23.757	Naphthalene, 2,3-dimethyl-	5.24	2-ring AHs
30	23.863	Naphthalene, 2,6-dimethyl-	2.97	2-ring AHs
31	24.275	Naphthalene, 2,3-dimethyl-	1.96	2-ring AHs
32	25.913	Naphthalene, 1,4,6-trimethyl-	0.94	2-ring AHs

33	26.395	4,6,8-Trimethylazulene	1.53	2-ring AHs
34	26.562	4,6,8-Trimethylazulene	2.16	2-ring AHs
35	26.898	Naphthalene, 1,4,6-trimethyl-	1.39	2-ring AHs
36	27.018	4,6,8-Trimethylazulene	1.64	2-ring AHs
37	28.178	Dodecane, 2-methyl-6-propyl-	1.55	Alkanes
38	30.451	Dodecane, 2-methyl-6-propyl-	2.22	Alkanes
39	30.574	9H-Fluorene, 2-methyl-	1.43	2-ring AHs
40	32.25	Phenanthrene	1.6	3-ring AHs
41	34.615	Phenanthrene, 1-methyl-	2.25	3-ring AHs
42	34.726	Phenanthrene, 1-methyl-	1.87	3-ring AHs
43	34.936	1H-Cyclopropa[l]phenanthrene,1a,9b-dihydro-	1.47	3-ring AHs
44	36.943	Phenanthrene, 2,5-dimethyl-	0.86	3-ring AHs
45	37.034	Phenanthrene, 2,5-dimethyl-	1.04	3-ring AHs
46	37.277	Phenanthrene, 2,5-dimethyl-	2.82	3-ring AHs
47	37.411	Phenanthrene, 2,5-dimethyl-	1.89	3-ring AHs
48	39.477	Phenanthrene, 2,3,5-trimethyl-	1.5	3-ring AHs
49	42.678	6,6-Diphenylfulvene	1.11	#N/A
50	46.544	Triphenylene, 2-methyl-	1.09	4-ring AHs

Table S5. Major components identified in tar from catalytic pyrolysis by CGY-SN.

Number	Retention time (min)	Name	Relative content (%)	Category
1	4.334	Toluene	2.13	1-ring AHs
2	6.259	Cyclohexane, 1,1,3-trimethyl-	3.41	Alkanes
3	7.194	o-Xylene	5.96	1-ring AHs
4	7.899	Benzene, 1,3-dimethyl-	1.53	1-ring AHs
5	10.143	Benzene, 1,2,3-trimethyl-	1.74	1-ring AHs
6	10.426	Benzene, 1,2,4-trimethyl-	1.89	1-ring AHs
7	11.223	Benzene, 1,2,4-trimethyl-	5.21	1-ring AHs
8	11.486	Undecane	1.24	Alkanes
9	12.087	Benzene, 1,2,4-trimethyl-	0.96	1-ring AHs
10	13.275	Benzene, 1-ethyl-2,3-dimethyl-	1.04	1-ring AHs
11	13.41	Phenol, 3-methyl-	1.9	Phenols
12	14.14	Phenol, 3-methyl-	4.54	Phenols
13	14.674	Undecane	1.52	Alkanes
14	16.093	1H-Indene, 2,3-dihydro-5-methyl-	1.02	1-ring AHs
15	16.291	Phenol, 2,5-dimethyl-	1.88	Phenols
16	16.358	Phenol, 2,6-dimethyl-	1.02	Phenols
17	16.976	Phenol, 2,3-dimethyl-	1.04	Phenols
18	17.222	Azulene	2.04	2-ring AHs
19	17.713	Tridecane	1.36	Alkanes
20	19.092	1H-Indene, 2,3-dihydro-1,6-dimethyl-	1.12	1-ring AHs
21	19.557	Phenol, 2,3,6-trimethyl-	1.03	Phenols
22	20.445	Naphthalene, 2-methyl-	6.57	2-ring AHs
23	20.569	Dodecane	1.39	Alkanes
24	20.862	Naphthalene, 1-methyl-	2.7	2-ring AHs
25	23.256	Dodecane	1.06	Alkanes
26	23.397	Naphthalene, 1,6-dimethyl-	2.17	2-ring AHs
27	23.447	Naphthalene, 2,6-dimethyl-	2.52	2-ring AHs
28	23.752	Naphthalene, 2,3-dimethyl-	4.85	2-ring AHs
29	23.864	Naphthalene, 2,6-dimethyl-	2.78	2-ring AHs
30	24.274	Naphthalene, 2,3-dimethyl-	1.82	2-ring AHs
31	26.393	4,6,8-Trimethylazulene	1.46	2-ring AHs
32	26.56	4,6,8-Trimethylazulene	1.83	2-ring AHs

33	26.896	Naphthalene, 1,4,6-trimethyl-	1.32	2-ring AHs
34	27.017	Naphthalene, 1,4,6-trimethyl-	1.54	2-ring AHs
35	27.958	1,1'-Biphenyl, 3,4'-dimethyl-	1.05	1-ring AHs
36	28.179	Dodecane, 2-methyl-6-propyl-	2.02	Alkanes
37	30.449	Decane, 1-bromo-2-methyl-	2.11	Alkanes
38	30.573	9H-Fluorene, 2-methyl-	1.4	2-ring AHs
39	32.251	Phenanthrene	1.46	3-ring AHs
40	34.618	Phenanthrene, 1-methyl-	2.11	3-ring AHs
41	34.726	Phenanthrene, 1-methyl-	1.71	3-ring AHs
42	34.941	1H-Cyclopropa[l]phenanthrene,1a,9b-dihydro-	1.26	3-ring AHs
43	37.025	Phenanthrene, 2,5-dimethyl-	1.08	3-ring AHs
44	37.274	Phenanthrene, 2,5-dimethyl-	2.6	3-ring AHs
45	37.41	Phenanthrene, 2,5-dimethyl-	1.83	3-ring AHs
46	39.476	Phenanthrene, 2,3,5-trimethyl-	1.27	3-ring AHs
47	40.966	Pyrene, 1-methyl-	0.94	4-ring AHs
48	42.677	Pyrene, 1,3-dimethyl-	1.2	4-ring AHs
49	44.693	Benz[a]anthracene	1.31	4-ring AHs
50	45.058	7,12-Dihydro-2-methylbenz[a]anthracene	1.1	4-ring AHs

Table S6. Major components identified in tar from catalytic pyrolysis by CGH.

Number	Retention time (min)	Name	Relative content (%)	Category
1	4.335	Toluene	1.55	1-ring AHs
2	6.259	Cyclohexane, 1,1,3-trimethyl-	2.95	Alkanes
3	7.194	o-Xylene	5.61	1-ring AHs
4	7.899	Benzene, 1,3-dimethyl-	1.44	1-ring AHs
5	10.143	Benzene, 1,2,3-trimethyl-	1.7	1-ring AHs
6	10.242	Benzene, 1,2,3-trimethyl-	0.91	1-ring AHs
7	10.425	Benzene, 1,2,4-trimethyl-	1.91	1-ring AHs
8	11.224	Benzene, 1,2,4-trimethyl-	5.3	1-ring AHs
9	11.486	Undecane	1.27	Alkanes
10	12.086	Benzene, 1,2,4-trimethyl-	0.99	1-ring AHs
11	12.504	Tetracyclo[3.3.1.0(2,8).0(4,6)]-non-2-ene	0.91	1-ring AHs
12	13.411	Phenol, 3-methyl-	1.99	Phenols
13	14.146	Benzene, 1-[(3-chloro-2-propenyl)oxy]-2-methyl-	4.01	Phenols
14	14.677	Undecane	1.49	Alkanes
15	16.094	1H-Indene, 2,3-dihydro-4-methyl-	1.13	1-ring AHs
16	16.292	Phenol, 2,5-dimethyl-	1.86	Phenols
17	16.358	Phenol, 2,6-dimethyl-	1.15	Phenols
18	16.978	Phenol, 2,3-dimethyl-	1.01	Phenols
19	17.223	Azulene	2.2	2-ring AHs
20	17.714	Undecane	1.27	Alkanes
21	19.558	Oxalic acid, heptyl 2-isopropylphenyl ester	1.26	#N/A
22	20.443	Naphthalene, 2-methyl-	6.72	2-ring AHs
23	20.568	Dodecane	1.37	Alkanes
24	20.86	Naphthalene, 1-methyl-	2.73	2-ring AHs
25	23.255	Dodecane	1.01	Alkanes
26	23.397	Naphthalene, 2,6-dimethyl-	2.14	2-ring AHs
27	23.444	Naphthalene, 2,6-dimethyl-	2.46	2-ring AHs
28	23.752	Naphthalene, 2,3-dimethyl-	4.92	2-ring AHs
29	23.861	Naphthalene, 2,6-dimethyl-	2.77	2-ring AHs
30	24.274	Naphthalene, 2,3-dimethyl-	1.83	2-ring AHs
31	26.392	Naphthalene, 1,4,6-trimethyl-	1.47	2-ring AHs
32	26.558	4,6,8-Trimethylazulene	2.08	2-ring AHs

33	26.898	Naphthalene, 1,4,6-trimethyl-	1.36	2-ring AHs
34	27.017	4,6,8-Trimethylazulene	1.6	2-ring AHs
35	27.311	3-(2-Methyl-propenyl)-1H-indene	0.86	1-ring AHs
36	27.813	Fluorene	1.22	2-ring AHs
37	28.179	Dodecane, 2-methyl-6-propyl-	1.38	Alkanes
38	30.451	3H-Benz[e]indene, 2-methyl-	2.09	Alkanes
39	30.571	9H-Fluorene, 2-methyl-	1.35	2-ring AHs
40	32.25	Phenanthrene	1.5	3-ring AHs
41	34.612	Anthracene, 9-methyl-	2.14	3-ring AHs
42	34.725	Anthracene, 9-methyl-	1.77	3-ring AHs
43	34.938	Naphtho[2,3-b]norbornadiene	1.46	3-ring AHs
44	37.042	Phenanthrene, 2,5-dimethyl-	1.05	3-ring AHs
45	37.273	Phenanthrene, 2,5-dimethyl-	2.54	3-ring AHs
46	37.409	Phenanthrene, 2,5-dimethyl-	2.56	3-ring AHs
47	39.474	Phenanthrene, 2,3,5-trimethyl-	1.35	3-ring AHs
48	42.678	Pyrene, 1,3-dimethyl-	1.56	4-ring AHs
49	46.542	Benz[a]anthracene, 7-methyl-	1.09	4-ring AHs
50	47.106	4b,10b-Dihydro-4b,10b-methanochrysene	1.71	4-ring AHs

Table S7. Major components identified in tar from catalytic pyrolysis by CGH-N.

Number	Retention time (min)	Name	Relative content (%)	Category
1	4.336	Toluene	2.16	1-ring AHs
2	6.26	Cyclohexane, 1,1,3-trimethyl-	2.96	Alkanes
3	6.911	Ethylbenzene	0.86	1-ring AHs
4	7.195	o-Xylene	5.44	1-ring AHs
5	7.242	o-Xylene	1.5	1-ring AHs
6	7.9	o-Xylene	1.66	1-ring AHs
7	8.204	Nonane	0.67	Alkanes
8	10.144	Benzene, 1,2,3-trimethyl-	2.16	1-ring AHs
9	10.244	Benzene, 1,2,3-trimethyl-	1.07	1-ring AHs
10	10.426	Benzene, 1,2,4-trimethyl-	2.25	1-ring AHs
11	11.224	Benzene, 1,2,4-trimethyl-	5.69	1-ring AHs
12	11.488	Undecane	1.03	Alkanes
13	12.087	Benzene, 1,2,4-trimethyl-	1.03	1-ring AHs
14	12.508	Tetracyclo[3.3.1.0(2,8).0(4,6)]-non-2-ene	1.21	1-ring AHs
15	13.276	Benzene, 2-ethyl-1,3-dimethyl-	0.92	1-ring AHs
16	13.409	Phenol, 3-methyl-	2.47	Phenols
17	14.142	Benzene, 1-[(3-chloro-2-propenyl)oxy]-2-methyl-	5.05	Phenols
18	14.676	Undecane	1.61	Alkanes
19	15.829	Benzene, 1-ethenyl-4-ethyl-	0.98	1-ring AHs
20	16.093	1H-Indene, 2,3-dihydro-4-methyl-	1.27	1-ring AHs
21	16.29	Phenol, 2,5-dimethyl-	2.22	Phenols
22	16.358	Phenol, 2,6-dimethyl-	1.29	Phenols
23	16.977	Phenol, 2,3-dimethyl-	1.08	Phenols
24	17.223	Azulene	2.25	2-ring AHs
25	17.3	1H-Indene, 2,3-dihydro-1,6-dimethyl-	0.45	1-ring AHs
26	17.714	Tridecane	1.22	Alkanes
27	20.445	Naphthalene, 2-methyl-	6.56	2-ring AHs
28	20.567	Dodecane	1.53	Alkanes
29	20.862	Naphthalene, 1-methyl-	2.86	2-ring AHs
30	23.083	Naphthalene, 1-ethyl-	0.25	2-ring AHs
31	23.256	Dodecane	0.96	Alkanes
32	23.392	Naphthalene, 2,6-dimethyl-	2.62	2-ring AHs

33	23.447	Naphthalene, 2,6-dimethyl-	2.31	2-ring AHs
34	23.755	Naphthalene, 2,3-dimethyl-	5.55	2-ring AHs
35	23.861	Naphthalene, 2,6-dimethyl-	3.06	2-ring AHs
36	25.785	Pentadecane	0.89	Alkanes
37	26.395	Naphthalene, 1,4,6-trimethyl-	1.75	2-ring AHs
38	26.557	4,6,8-Trimethylazulene	2.33	2-ring AHs
39	26.894	Naphthalene, 1,4,6-trimethyl-	1.54	2-ring AHs
40	27.017	4,6,8-Trimethylazulene	1.77	2-ring AHs
41	28.182	Dodecane, 2-methyl-6-propyl-	1.63	Alkanes
42	30.449	Dodecane, 2-methyl-6-propyl-	2.13	Alkanes
43	30.574	9H-Fluorene, 2-methyl-	1.54	2-ring AHs
44	32.251	Phenanthrene	1.55	3-ring AHs
45	32.605	Hexadecane	0.73	Alkanes
46	34.613	Anthracene, 9-methyl-	2.42	3-ring AHs
47	34.729	Phenanthrene, 1-methyl-	1.95	3-ring AHs
48	35.082	1H-Cyclopropa[l]phenanthrene,1a,9b-dihydro-	0.84	3-ring AHs
49	35.199	Anthracene, 9-methyl-	0.85	3-ring AHs
50	37.275	Phenanthrene, 2,5-dimethyl-	1.9	3-ring AHs

Table S8. Major components identified in tar from catalytic pyrolysis by CGH-ON.

Number	Retention time (min)	Name	Relative content (%)	Category
1	4.336	Toluene	1.49	1-ring AHs
2	6.261	Cyclohexane, 1,1,3-trimethyl-	2.52	Alkanes
3	7.197	o-Xylene	4.13	1-ring AHs
4	7.242	o-Xylene	1.19	1-ring AHs
5	7.901	o-Xylene	1.36	1-ring AHs
6	10.146	Benzene, 1,2,3-trimethyl-	1.74	1-ring AHs
7	10.429	Benzene, 1,2,4-trimethyl-	1.9	1-ring AHs
8	11.226	Benzene, 1,2,4-trimethyl-	5.38	1-ring AHs
9	11.49	Undecane	1.11	Alkanes
10	12.09	Benzene, 1,2,4-trimethyl-	0.99	1-ring AHs
11	12.513	Tetracyclo[3.3.1.0(2,8).0(4,6)]-non-2-ene	0.93	1-ring AHs
12	13.277	Benzene, 2-ethyl-1,3-dimethyl-	1.09	1-ring AHs
13	13.408	Phenol, 3-methyl-	2.2	Phenols
14	14.14	Phenol, 3-methyl-	4.47	Phenols
15	14.677	Undecane	1.53	Alkanes
16	16.094	1H-Indene, 2,3-dihydro-4-methyl-	1.1	1-ring AHs
17	16.288	Phenol, 2,5-dimethyl-	2.13	Phenols
18	16.35	Phenol, 2,6-dimethyl-	1.25	Phenols
19	16.974	Phenol, 2,3-dimethyl-	1.17	Phenols
20	17.224	Azulene	2.49	2-ring AHs
21	17.716	Tridecane	1.31	Alkanes
22	20.45	Naphthalene, 2-methyl-	6.48	2-ring AHs
23	20.572	Dodecane	1.33	Alkanes
24	20.866	Naphthalene, 1-methyl-	2.79	2-ring AHs
25	23.26	Dodecane	1.04	Alkanes
26	23.402	Naphthalene, 2,6-dimethyl-	2.36	2-ring AHs
27	23.449	Naphthalene, 1,6-dimethyl-	2.38	2-ring AHs
28	23.761	Naphthalene, 2,3-dimethyl-	5.2	2-ring AHs
29	23.868	Naphthalene, 2,6-dimethyl-	2.94	2-ring AHs
30	24.277	Naphthalene, 2,3-dimethyl-	1.9	2-ring AHs
31	25.924	Naphthalene, 1,4,6-trimethyl-	1	2-ring AHs
32	26.401	Naphthalene, 1,4,6-trimethyl-	1.59	2-ring AHs

33	26.563	4,6,8-Trimethylazulene	2.16	2-ring AHs
34	26.901	Naphthalene, 1,4,6-trimethyl-	1.42	2-ring AHs
35	27.022	4,6,8-Trimethylazulene	1.73	2-ring AHs
36	27.958	1,1'-Biphenyl, 3,4'-dimethyl-	1.2	1-ring AHs
37	28.188	Dodecane, 2-methyl-6-propyl-	2.1	Alkanes
38	30.453	Decane, 1-bromo-2-methyl-	2.18	Alkanes
39	30.577	9H-Fluorene, 2-methyl-	1.46	2-ring AHs
40	32.256	Phenanthrene	1.55	3-ring AHs
41	34.622	Phenanthrene, 1-methyl-	2.23	3-ring AHs
42	34.733	Phenanthrene, 1-methyl-	1.73	3-ring AHs
43	34.944	1H-Cyclopropa[l]phenanthrene,1a,9b-dihydro-	1.41	3-ring AHs
44	37.04	Phenanthrene, 2,5-dimethyl-	0.97	3-ring AHs
45	37.284	Phenanthrene, 2,5-dimethyl-	2.82	3-ring AHs
46	37.425	Phenanthrene, 2,5-dimethyl-	1.99	3-ring AHs
47	39.484	Phenanthrene, 2,3,5-trimethyl-	1.36	3-ring AHs
48	40.977	Pyrene, 1-methyl-	0.98	4-ring AHs
49	44.742	Benz[a]anthracene	1.03	4-ring AHs
50	46.549	Benz[a]anthracene, 7-methyl-	1.19	4-ring AHs

Table S9. Major components identified in tar from catalytic pyrolysis by CGH-SN.

Number	Retention time (min)	Name	Relative content (%)	Category
1	4.339	Toluene	3.27	1-ring AHs
2	6.265	Cyclohexane, 1,1,3-trimethyl-	4.76	Alkanes
3	6.915	Ethylbenzene	0.91	1-ring AHs
4	7.199	o-Xylene	5.57	1-ring AHs
5	7.242	o-Xylene	1.79	1-ring AHs
6	7.903	o-Xylene	1.86	1-ring AHs
7	10.147	Benzene, 1-ethyl-3-methyl-	2	1-ring AHs
8	10.248	Benzene, 1-ethyl-3-methyl-	1.06	1-ring AHs
9	10.429	Benzene, 1,2,4-trimethyl-	2.15	1-ring AHs
10	11.229	Benzene, 1,2,4-trimethyl-	5.79	1-ring AHs
11	11.491	Undecane	1.41	Alkanes
12	12.09	Benzene, 1,2,4-trimethyl-	1.01	1-ring AHs
13	12.513	Tetracyclo[3.3.1.0(2,8).0(4,6)]-non-2-ene	1.03	1-ring AHs
14	13.281	Benzene, 2-ethyl-1,3-dimethyl-	1.12	1-ring AHs
15	13.414	Phenol, 3-methyl-	2.03	Phenols
16	14.142	Benzene, 1-[(3-chloro-2-propenyl)oxy]-2-methyl-	4.05	Phenols
17	14.677	Undecane	1.58	Alkanes
18	16.095	1H-Indene, 2,3-dihydro-4-methyl-	1.08	1-ring AHs
19	16.293	Phenol, 2,5-dimethyl-	1.74	Phenols
20	16.358	Phenol, 2,6-dimethyl-	1.04	Phenols
21	16.977	Phenol, 2,3-dimethyl-	0.98	Phenols
22	17.224	Azulene	1.88	2-ring AHs
23	17.715	Tridecane	1.34	Alkanes
24	20.446	Naphthalene, 2-methyl-	5.78	2-ring AHs
25	20.571	Dodecane	1.38	Alkanes
26	20.863	Naphthalene, 1-methyl-	2.46	2-ring AHs
27	23.257	Dodecane	0.97	Alkanes
28	23.401	Naphthalene, 2,6-dimethyl-	2.12	2-ring AHs
29	23.449	Naphthalene, 2,6-dimethyl-	2.1	2-ring AHs
30	23.754	Naphthalene, 2,3-dimethyl-	4.58	2-ring AHs
31	23.863	Naphthalene, 2,6-dimethyl-	2.48	2-ring AHs
32	24.278	Naphthalene, 2,3-dimethyl-	1.6	2-ring AHs

33	26.397	Naphthalene, 1,4,6-trimethyl-	1.41	2-ring AHs
34	26.562	4,6,8-Trimethylazulene	1.99	2-ring AHs
35	26.899	Naphthalene, 1,4,6-trimethyl-	1.32	2-ring AHs
36	27.022	3-(2-Methyl-propenyl)-1H-indene	1.49	2-ring AHs
37	28.183	Dodecane, 2-methyl-6-propyl-	1.64	Alkanes
38	30.451	Sulfurous acid, hexyl octyl ester	2	Alkanes
39	30.578	9H-Fluorene, 2-methyl-	1.3	2-ring AHs
40	32.257	Phenanthrene	1.25	3-ring AHs
41	34.62	Anthracene, 9-methyl-	1.44	3-ring AHs
42	34.729	Anthracene, 9-methyl-	1.52	3-ring AHs
43	34.946	1H-Cyclopropa[l]phenanthrene,1a,9b-dihydro-	1.3	3-ring AHs
44	37.025	Phenanthrene, 2,5-dimethyl-	1.04	3-ring AHs
45	37.276	Phenanthrene, 2,5-dimethyl-	2.41	3-ring AHs
46	37.413	Phenanthrene, 2,5-dimethyl-	2.43	3-ring AHs
47	39.483	Phenanthrene, 2,3,5-trimethyl-	1.34	3-ring AHs
48	40.97	11H-Benzo[b]fluorene	1.03	4-ring AHs
49	42.679	Pyrene, 1,3-dimethyl-	1.29	4-ring AHs
50	46.551	Benz[a]anthracene, 7-methyl-	0.89	4-ring AHs
